# In vitro characterization on the role of *APOE* polymorphism in human hippocampal neurogenesis

**DOI:** 10.1002/hipo.23502

**Published:** 2023-01-28

**Authors:** Hyunah Lee, Jack Price, Deepak P. Srivastava, Sandrine Thuret

**Affiliations:** ^1^ Department of Basic and Clinical Neuroscience, Institute of Psychiatry, Psychology and Neuroscience King's College London London UK; ^2^ MRC Centre for Neurodevelopmental Disorders King's College London London UK

**Keywords:** apolipoprotein E4, hippocampus, induced pluripotent stem cells, neurogenesis, transcriptome

## Abstract

Hippocampal neurogenesis (HN) is considered an important mechanism underlying lifelong brain plasticity, and alterations in this process have been implicated in early Alzheimer's disease progression. *APOE* polymorphism is the most common genetic risk factor for late‐onset Alzheimer's disease where the ε4 genotype is associated with a significantly earlier disease onset compared to the neutral ε3 allele. Recently, *APOE* has been shown to play an important role in the regulation of HN. However, the time‐dependent impact of its polymorphism in humans remains elusive, partially due to the difficulties of studying human HN in vivo. To bridge this gap of knowledge, we used an in vitro cellular model of human HN and performed a time course characterization on isogenic induced pluripotent stem cells with different genotypes of *APOE*. We found that *APOE* itself was more highly expressed in ε4 at the stem cell stage, while the divergence of differential gene expression phenotype between ε4 and ε3 became prominent at the neuronal stage of differentiation. This divergence was not associated with the differential capacity to generate dentate gyrus granule cell‐like neurons, as its level was comparable between ε4 and ε3. Transcriptomic profiling across different stages of neurogenesis indicated a clear “maturation of functional neurons” phenotype in ε3 neural progenitors and neurons, while genes differentially expressed only in ε4 neurons suggested potential alterations in “metabolism and mitochondrial function.” Taken together, our in vitro investigation suggests that *APOE* ε4 allele can exert a transcriptome‐wide effect at the later stages of HN, without altering the overall level of neurogenesis per se.

## 1 INTRODUCTION

Hippocampal neurogenesis (HN) in the mammalian brain occurs throughout life in the subgranular zone (SGZ) of the dentate gyrus (DG) (Drew et al., 2013). The principal excitatory neurons born from HN are denoted DG granule cells (DGCs). They receive input from the entorhinal cortex and send output to the CA3 region of the hippocampus, playing a pivotal role in memory and mood regulation (Kempermann et al., 2015). For newborn neurons to be produced through HN, “quiescent stem cells” residing in the SGZ (type‐1 or radial glia‐like stem cells expressing markers like Paired Box 6 [PAX6], Maekawa et al., 2005, Hes family BHLH transcription factor 5 [HES5], Jin et al., 2003, and achaete‐scute family BHLH transcription factor 1 [ASCL1], Andersen et al., 2014), first need to become “transient intermediate progenitor cells” (type‐2 cells expressing markers like eomesodermin [EOMES/TBR2], Hodge et al., 2008, empty spiracles homeobox 2 [EMX2], Mariani et al., 2012, and forkhead box G1 [FOXG1], Shen et al., 2006). These cells will then differentiate into “neuroblasts” (type‐3 cells expressing markers like doublecortin [DCX], Jessberger et al., 2005, neuronal differentiation 1 [NEUROD1], Kuwabara et al., 2009, and microtubule associated protein tau [MAPT], Llorens‐Martin et al., 2012), which eventually exit the cell cycle and begin to mature into “DGCs” marked by increased expression of calretinin, calbindin, RE1 silencing transcription factor (REST), and prospero homeobox 1 (PROX1) (Hsieh, 2012).

Evidence suggests that the expression of notable cellular markers is highly conserved across species; for example, nestin, sex determining region Y‐Box 2 (SOX2), DCX, polysialylated neuronal cell adhesion molecule (PSA‐NCAM), and PROX1 are highly expressed in similar cell types across rodent, primate, and human HN (Charvet & Finlay, 2018; Miller et al., 2013). Although the persistence of HN throughout life in humans has been recently disputed (Cipriani et al., 2018; Sorrells et al., 2018), the majority of the current literature (Kempermann et al., 2018) suggests that HN is a highly conserved phenomenon, robustly observed across many mammalian species, and that newborn cells generated through HN are critical for hippocampus‐dependent learning and memory (Deng et al., 2010; Gonçalves et al., 2016).

Alzheimer's disease (AD) is the most common cause of dementia affecting more than 50 million people worldwide (Prince et al., 2015). In contrast to early‐onset AD, which is caused by autosomal‐dominant mutations in genes such as amyloid precursor protein (APP), presenilin 1 (PSEN1), and presenilin 2 (PSEN2), late‐onset AD occurs “spontaneously” with age (Van Cauwenberghe et al., 2016). An overwhelming majority of AD cases is late‐onset (approximately 95%), and both early and late‐onset AD share similar clinical phenotypes (i.e., severe memory loss and cognitive decline accompanied by changes in mood and behavior) (Reitz et al., 2011) and similar biological hallmarks (i.e., β‐amyloid plaques, neurofibrillary tangles of hyperphosphorylated tau, and progressive neurodegeneration in multiple brain regions) (Serrano‐Pozo et al., 2011).

HN is significantly reduced in post‐mortem human AD brains (Ekonomou et al., 2015; Moreno‐Jiménez et al., 2019; Tobin et al., 2019), and alterations in this process are one of the earliest changes observed in AD (Unger et al., 2016). HN can be a promising therapeutic target for the prevention and delay of AD onset, because it is influenced by many environmental factors that contribute to AD risk (Toda et al., 2019). However, contradictory findings on the direction and magnitude of change in HN make it difficult to pinpoint the exact approach that should be taken. Some studies show that HN is reduced in AD (Wang et al., 2004; Zhang et al., 2007), while others report that the expression of neurogenic markers is “increased” in AD (Jin, Galvan, et al., 2004; Jin, Peel, et al., 2004). One way to explain such discrepancy is to think of “increased” neurogenesis as a potential compensatory mechanism triggered to replenish the ongoing neuronal loss, while survival and maturation for the newborn neurons can ultimately result in failure (Chen et al., 2008; Sun et al., 2009). In line with this notion, transcriptomic studies on human AD brains collectively indicate that the expression of “early” neurogenic markers (i.e., markers for neural progenitor cells [NPCs] and proliferation) is “up‐regulated,” whereas that of “late” neurogenic markers (i.e., markers for maturation and survival) is “down‐regulated” in AD (Gatt et al., 2019). Further investigations in animal studies with a higher temporal resolution would be able to clarify whether this observation in humans can be generalized to animal models. Nevertheless, a tentative summary of the existing literature can be made as follows: the overall level of HN is reduced in AD, potentially due to the failure of maturation and integration of newborn neurons, while NPCs might proliferate more over the course of disease progression as a compensatory mechanism.

The most common genetic risk factor for late‐onset AD is Apolipoprotein E (APOE) polymorphism (Shen & Jia, 2016; Van Cauwenberghe et al., 2016). Two single nucleotide polymorphisms (SNPs) at the protein‐coding region of *APOE* (exon 4) make up the following genotypes: epsilon 3, 2, and 4 (ɛ3, ɛ2, and ɛ4). The most common isoform of *APOE* is ɛ3, which has thymine (T) and cytosine (C) at the rs429358 and rs7412 SNP regions, respectively. In contrast, T at both regions make up the ɛ2 isoform, and C at both regions make up the ɛ4 isoform (Liu et al., 2013). It is the ɛ4 allele, which is considered the most common genetic risk factor of late‐onset AD (Rebeck et al., 1993), and the frequency of ɛ4 is higher in people diagnosed with AD compared to healthy controls. The effects of ɛ4 on elevated AD risk are allele dose dependent, where the odds ratio can be increased up to 14.9 for people with homozygous ɛ4 alleles (ε2/ε4 = 2.6, ε3/ε4 = 3.2) (Farrer et al., 1997; Sando et al., 2008). Notably, ɛ4 carriers develop AD at an earlier age (Corder et al., 1993; Farrer et al., 1997; Rebeck et al., 1993), and they also have a higher rate of progression from MCI to AD (Bonham et al., 2016).

Interestingly, postnatal neural stem/progenitor cells in the adult DG of mice express high levels of *APOE* (Gilley et al., 2011) and is essential in maintaining the number of these cells throughout adulthood (Yang et al., 2011). Studies on injury‐induced HN have shown that ɛ4 allele and knockout behave similarly, in which they can both impair dendritic arborization and reduce spine density of adult‐born DGCs (Hong et al., 2016; Tensaouti et al., 2018). However, in injury‐free conditions, impaired maturation of adult‐born DGCs is more specific to ɛ4 and is not evident in knockout mice (Li et al., 2009). Moreover, ɛ4 hippocampal progenitors tend to proliferate more than ɛ3 progenitors, while the lack of *APOE* is more strongly characterized by increased gliogenesis (Li et al., 2009). Taken together, the evidence generated from various mouse studies suggests that *APOE* plays an important role in the overall maintenance of the hippocampal neurogenic niche, while ɛ4 allele is associated more specifically with abnormal proliferation of progenitor cells and failure of maturation in newborn neurons. Importantly, our understanding on how *APOE* genotype affects HN at the cellular level is yet to be obtained in humans, partially due to the difficulties of studying HN dynamics in live subjects.

While there is currently no in vitro model that specifically recapitulates “adult” human HN, evidence suggests that “embryonic” and “postnatal” HN follow a similar pattern of precursor expansion and neuroblast maturation once the radial glia‐like stem cells become activated (Espósito et al., 2005; Hochgerner et al., 2018; Urbán & Guillemot, 2014). Previously, Yu and colleagues developed a differentiation paradigm that recapitulates key developmental events that occur in both embryonic and postnatal HN, demonstrating that human pluripotent stem cells can be differentiated into hippocampal DGC‐like cells expressing PROX1 using this protocol (Yu et al., 2014). In this study, we used a similar in vitro model of HN based on Yu and colleagues' method that can generate DGC‐like cells from human induced pluripotent stem cells (iPSCs). We aimed to characterize the phenotypes of HN according to various *APOE* genotypes at the cellular level using this model and isogenic human iPSCs.

## 2 METHODS

### 2.1 Cell lines

Isogenic iPSCs (male origin) were obtained from the European Bank for induced pluripotent Stem Cells (EBiSC). Full data on reprogramming and characterization of BIONi010‐C‐2 (ɛ3 genotype, denoted E3 in this article; RRID:CVCL_II81), BIONi010‐C‐4 (ɛ4 genotype, denoted E4 in this article; RRID:CVCL_II83), BIONi010‐C‐6 (ɛ2 genotype, denoted E2 in this article; RRID:CVCL_II85), and BIONi010‐C‐3 (*APOE* knockout, denoted KO in this article; RRID:CVCL_II82) lines can be found in the report by Schmid and colleagues (Schmid et al., 2019). A corrigendum to this article shows that E3, E4, and E2 lines have only one functional ɛ3, ɛ4, ɛ2 allele of *APOE* expressed with the correct genotype, respectively; the KO line does not express any functional *APOE* (Schmid et al., 2020).

### 2.2 Maintenance of iPSCs

All cells were regularly tested for mycoplasma and certified mycoplasma free. All iPSCs were maintained in StemFlex™ Medium (Thermo Fisher, Cat# A3349401) without antibiotics at 37°C, 5% CO_2_, 5% O_2_ conditions using sterile six‐well NUNC™ plates (Thermo Fisher, Cat# 140675) coated with Geltrex™ (Thermo Fisher, Cat# A1413302). Geltrex™ solution was prepared in 2–4°C Dulbecco's Modified Eagle's Medium/Nutrient Mixture F‐12 Ham (DMEM/F12) (Sigma Aldrich, Cat# D6421) according to the manufacturer's instructions, and all plates were coated 1 h before use.

Maintenance passaging of iPSCs was done with Versene‐ethylenediaminetetraacetic acid (EDTA) solution (Lonza, Cat# BE17‐711 E) according to the manufacturer's instructions. Cells at 70%–80% confluence were washed with 1 ml/well room temperature Hank's Balanced Salt Solution (HBSS) (Thermo Fisher, Cat# 14170‐161) and incubated with 1 ml/well room temperature Versene for 3–4 min. After aspirating Versene from each well, cells were detached gently (2–3 wells at a time) with 5–6 ml of room temperature StemFlex™, using 5 ml or 10 ml stripettes. Detached cells were collected in new 50 ml conical tube(s) and were gently broken down with 5 ml or 10 ml stripettes until the size of cell clumps were reduced to that of “dots” when viewed with unaided eyes. Passaging ratio was kept between 1:6 and 1:18 depending on the experimental requirements. Spontaneously differentiated iPSC colonies were regularly cleaned prior to passaging with sterile aspirator pipettes with 10/20 μl pipette tips inserted at the end. Time spent outside the incubator for cleaning was always kept under 5 min.

### 2.3 Directed differentiation of iPSCs to NPCs

#### 2.3.1 Replating iPSCs

**FIGURE 1:**
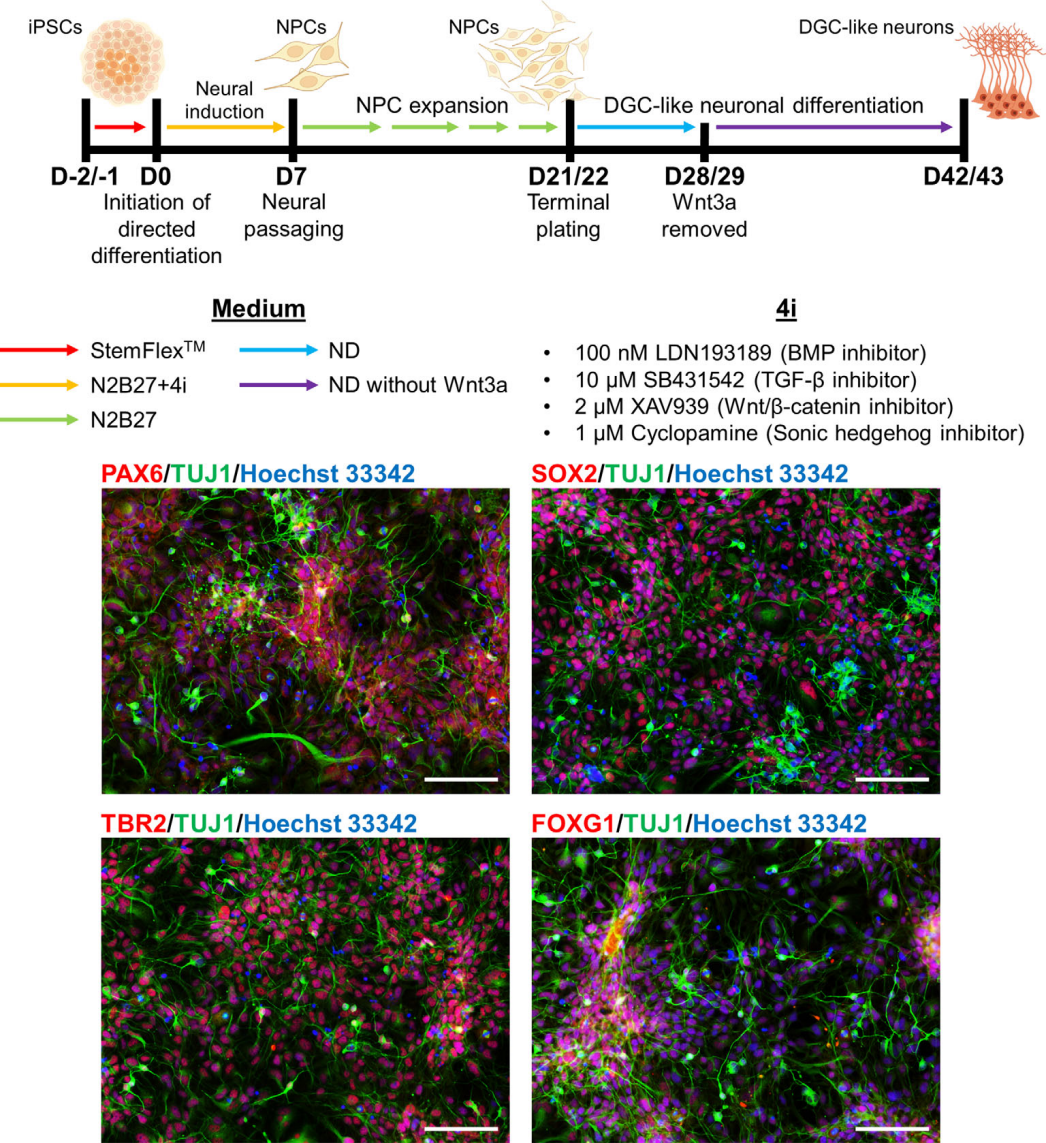
Directed differentiation of human induced pluripotent stem cells (iPSCs) to neural progenitors (NPCs) and dentate gyrus granule cell (DGC)‐like neurons. Top: Schematic diagram of directed differentiation protocol. Human iPSCs were replated 2 or 1 day(s) prior to the initiation of directed differentiation (D‐2/‐1; red arrow indicating the use of StemFlexTM medium). After 7 days of neural induction in N2B27 + 4i medium (yellow arrow), NPCs were expanded until Day 21/22 in N2B27 medium (light green arrow). Upon terminal plating, NPCs were differentiated to DGC‐like neurons. ND medium was used for the first 7 days until Day 28/29 (light blue arrow), and Wnt3a was removed afterward until Day 42/43 (purple arrow). The four inhibitors used for neural induction period (4i) are shown on the bottom right. Bottom: Representative images of Day 30 NPCs expressing transcription factors important for hippocampal cell fate determination (PAX6, SOX2, TBR2, FOXG1), and a neuron‐specific marker (TUJ1). Nuclei are counterstained with Hoechst 33342. Scale bar 100 μm

All stages of directed differentiation were performed in sterile six‐well NUNC™ plates except for terminal plating for DGC‐like neuronal differentiation. A schematic diagram summarizing the procedure is shown in Figure 1 (top panel). To replate iPSC colonies for directed differentiation, cells were first maintained to reach 70%–80% confluence and then lifted with 1 ml/well room temperature Versene. After aspirating the Versene, 1 ml/well room temperature StemFlex™ was introduced to all wells, and iPSC colonies were gently but swiftly scraped off with sterile cell scrapers (wedge facing the cells). Lifted colonies were then collected in new 50 ml conical tube(s) with either 5 ml or 10 ml stripettes. The colonies were gently pipetted up and down with a P1000 until they were broken down to small granules that were still visible to unaided eyes. Cells were then transferred to new Geltrex™ plates at a passaging ratio of 3:2, so that the confluence could reach near 100% in 24–48 hReplated iPSCs were incubated at 37°C, 5% CO_2_, 5% O_2_ conditions, and if confluence was not near 100% in 24 h, medium was changed to fresh StemFlex™ (3 ml/well) and incubated for an additional 24 h. Replated iPSCs that failed to reach near 100% confluence after 48 h were discarded and did not proceed to directed differentiation.

#### 2.3.2 Initiation of directed differentiation

Directed differentiation of replated iPSCs was initiated (Day 0) by changing the following culture conditions. StemFlex™ was replaced with N2B27 + 4i medium. This was a 1:1 mixture of N‐2 medium (DMEM/F12 supplemented with 1X GlutaMAX™ [Thermo Fisher, Cat# 35050–061] and 1X N‐2 [Thermo Fisher, Cat# 17502–048] and B‐27 medium [Neurobasal® medium; Thermo Fisher, Cat# 21103–049] supplemented with 1X GlutaMAX™ and 1X B‐27 minus vitamin A [Thermo Fisher, Cat# 12587–010]) **(**N2B27 medium**)**, containing 2 μM XAV939 (Sigma Aldrich, Cat# X3004) (Wnt/β‐catenin signaling pathway inhibitor), 10 μM SB431542 (Sigma Aldrich, Cat# S4317) (TGF‐β signaling pathway inhibitor), 100 nM LDN193189 (Sigma Aldrich, Cat# SML0559) (BMP signaling pathway inhibitor), and 1 μM Cyclopamine (LC Laboratories, Cat# C‐8700) (Sonic hedgehog signaling pathway inhibitor). N2B27 + 4i medium was changed every 24 h for additional 6 days (2 ml/well). Moreover, the O_2_ conditions were elevated from 5% to 20%.

#### 2.3.3 NPC expansion

On Day 7 of directed differentiation, the first neural passaging was done at 1:1 ratio. Cells were washed with 1 ml/well room temperature HBSS and incubated with 1 ml/well 4°C Accutase (Thermo Fisher, Cat# A11105‐01) for 3–4 min. Dissociated cells were collected into new 15 ml conical tube(s) that already contained room temperature DMEM/F12 at twice the volume of Accutase used. Importantly, the collection of cells in Accutase into conical tube(s) was done with no more than five times of pipetting with a P1000 to ensure passaging of cells in “small clumps” rather than “single cells.” Remaining clumps of cells in the plate that were not lifted at the first attempt were all collected with 1 ml/well room temperature DMEM/F12 using a P1000, and they were pooled together with the cells collected at the first attempt. Cells were centrifuged at 900 revolutions per minute (RPM) for 2 min twice. Prior to the second centrifugation, cells were resuspended/washed with DMEM/F12 at the same volume of Accutase used to completely remove traces of Accutase. During resuspension, extra care was taken not to break up the cells into single cells. Cells were plated on new Geltrex™ plates in N2B27 + 4i supplemented with 10 μM Y‐27632 (Sigma Aldrich, Cat# Y0503). Medium was changed the following day to N2B27 without Y‐27632 (Day 8). On Day 12, the second neural passaging was done at 1:1 ratio. The procedure was identical to the first neural passaging, except for the cells being plated in N2B27 supplemented with 10 μM Y‐27632. Medium was changed every 24 h (2 ml/well).

On Day 15/16, the third neural passaging for NPC expansion was done at 2:3 ratio. Cells were washed with 1 ml/well room temperature HBSS and incubated with 1 ml/well 37°C Accutase for 3–4 min. The handling restrictions applied at the first and second neural passaging to ensure passaging of cells in “small clumps” were all lifted. Gentle and persistent pipetting with P1000 was done to passage in single cells as much as possible. Once the cells were collected in new 15 ml conical tube(s) containing DMEM/F12 (as described above for the first and second neural passaging), cells were centrifuged at 1250 RPM for 2 min twice. Prior to the second centrifugation, cells were resuspended/washed with DMEM/F12 at the same volume of Accutase used to completely remove traces of Accutase. Cells were plated on new Geltrex™ plates in N2B27 supplemented with 10 μM Y‐27632. Medium was changed the next day to NPC medium without Y‐27632 (Day 16/17). On Day 18/19, the final neural passaging for NPC expansion was done at 2:3 ratio. The procedure was identical to the third neural passaging. Medium was changed every 24 h (2 ml/well).

#### 2.3.4 Terminal plating for DGC‐like neuronal differentiation

For DGC‐like neuronal differentiation, NPCs expanded as described above were terminally plated on either sterile 6‐well or 96‐well NUNC™ plates coated with poly‐l‐ornithine (Sigma Aldrich, Cat# P3655) and laminin (Sigma Aldrich, Cat# L2020) (POL) (Day 20/21). POL plates were prepared by incubating each well with 100 μg/ml poly‐ornithine for 3 h at 37°C (1.5 ml/well for 6‐well plates and 50 μl/well for 96‐well plates) followed by 20 μg/ml laminin overnight at 37°C (2 ml/well for 6‐well plates and 75 μl/well for 96‐well plates). Terminal plating procedure was identical to the third and final neural passaging, except for the passaging ratio, which was at 1:6 (or 20,000 cells/cm^2^).

On the next day of terminal plating (Day 21/22), DGC‐like neuronal differentiation was initiated by replacing N2B27 containing 10 μM Y‐27632 with neuronal differentiation medium (ND medium) made up with B‐27 medium containing 1 μg/ml laminin, 200 nM ascorbic acid (AA2P) (Sigma Aldrich, Cat# A4403), 1 mM dibutyryl cyclic adenosine monophosphate (dbcAMP) (Sigma Aldrich, Cat# D0627), 20 ng/mL brain‐derived neurotrophic factor (BDNF) (Peprotech, Cat# 450‐02), and 20 ng/ml WNT3A (R&D systems, Cat# 5036‐WN). ND medium was changed every 24 h for 7 days. WNT3A was removed on D28/29, and cells were differentiated for 14 more days until Day 42/43. Representative images of D30 cells are shown in Figure 1 (bottom panel).

### 2.4 Gene expression analysis

All cells harvested for gene expression analysis were cultured in six‐well plates. Total RNA was extracted using the TRIzol® reagent (Thermo Fisher, Cat# 15596026) according to manufacturer's instructions. Reverse transcription of total RNA into complementary DNA (cDNA) was performed using SuperScript® III First‐Strand Synthesis System (Thermo Fisher, Cat# 18080051) on S1000 Thermal Cycler (Bio‐Rad) according to manufacturer's instructions. cDNA for 1–5 μg of total RNA were synthesized per reaction. The reverse transcription product was diluted with DEPC‐treated water to achieve final concentration of 5 ng/μl total RNA converted to cDNA for real‐time semi‐quantitative polymerase chain reaction (qPCR). qPCR was performed with 5X HOT FIREPol® EvaGreen® qPCR Mix (Solis Biodyne, Cat# 08‐24‐00001) on either MJ Research PTC‐200 Thermal Cycler (Bio‐Rad) or Applied Biosystems QuantStudio 7 Flex Real‐Time PCR System (Thermo Fisher) according to manufacturer's instructions. HOT FIREPol® DNA Polymerase was activated first at 95°C for 15 min, followed by 45 cycles of (1) denaturation of cDNA at 95°C for 30 s, (2) annealing of primers at 60°C for 30 s, and (3) elongation at 72°C for 30 s. Relative expression of each gene was calculated by normalizing its C_T_ values to that of glyceraldehyde‐3‐phosphate dehydrogenase (GAPDH). List of primers is provided in Table 1.

**TABLE 1:** List of primers used in gene expression analysis

Gene	Forward	Reverse
*PAX6*	GCC CTC ACA AAC ACC TAC AG	TCA TAA CTC CGC CCA TTC AC
*SOX2*	AGC TAC AGC ATG ATG CAG GA	GGT CAT GGA GTT GTA CTG CA
*EMX2*	AGG GAC GCA CCA TAT TAA CC	CAC CTC TCC CTG TCT CTT TTG
*FOXG1*	AGA AGA ACG GCA AGT ACG AGA	TGT TGA GGG ACA GAT TGT GGC
*NEUROD1*	CCA GGG TTA TGA GAC TAT CAC TG	TCC TGA GAA CTG AGA CAC TCG
*MAP2*	CAG GAG ACA GAG ATG AGA ATT CC	CAG GAG TGA TGG CAG TAG AC
*DCX*	TCA GGG AGT GCG TTA CAT TTA C	GTT GGG ATT GAC ATT CTT GGT G
*PROX1*	GAC TTT GAG GTT CCA GAG AGA	TGT AGG CAG TTC GGG GAT TTG
*APOE*	GTT GCT GGT CAC ATT CCT GG	GCA GGT AAT CCC AAA AGC GAC
*GAPDH*	AGC CTC AAG ATC ATC AGC AA	CTG TGG TCA TGA GTC CTT CC

### 2.5 Immunocytochemistry

All cells used for immunocytochemistry (ICC) were cultured in 96‐well plates. Cells were fixed in 4% paraformaldehyde, permeabilized with 0.1% Triton™ X‐100 in 1X Tris‐buffered saline (TBS) for 15–30 min and then blocked with 5% normal donkey serum in TBS for 30 min. Primary antibodies were incubated at 4°C overnight followed by three washings with TBS. Secondary antibodies conjugated with fluorescent dyes were incubated at room temperature for 1 h followed by two washings with TBS. Nuclei were stained with 5 μg/ml Hoechst® 33,342 solution (Thermo Fisher, Cat# H3570) for 30 s immediately prior to imaging, and cells were washed with TBS two times after nuclear staining. All primary antibodies were diluted in 5% normal donkey serum in TBS, secondary antibodies in 1% normal donkey serum in TBS, and Hoechst® 33342 solution in TBS. All washings were done at 100 μl volume, and all incubations of antibodies and nuclear staining were done at 50 μl volume. Inverted epifluorescence microscopy was carried out with Olympus IX70 Inverted Fluorescence Microscope (RRID:SCR_018604) connected to AxioVision Imaging System (RRID:SCR_002677). List of antibodies is provided in Table 2.

**TABLE 2:** List of antibodies used in immunocytochemistry

	Dilution	Diluting solution	Host	Clonality	Company	Cat#; RRID
PAX6	1:200	5% Donkey serum (TBS^a^)	Rabbit	Poly	BioLegend	901301; AB_2565003
SOX2	1:100		Goat	Poly	Santa Cruz	sc‐17320; AB_2286684
TBR2	1:250		Rabbit	Poly	Abcam	ab23345; AB_778267
FOXG1	1:500		Rabbit	Poly	Abcam	ab18259; AB_732415
TUBB3 [TUJ1]	1:500		Mouse	Mono	BioLegend	801202; AB_10063408
MAP2 [HM‐2]	1:500		Mouse	Mono	Abcam	ab11267; AB_297885
PROX1	1:500		Rabbit	Poly	Abcam	ab101851; AB_10712211
Ki67 [8D5]	1:500		Mouse	Mono	Cell Signaling	9449; AB_2797703
CC3 (Asp175) [5A1E]	1:500		Rabbit	Mono	Cell Signaling	9664; AB_2070042
anti‐Mouse IgG Alexa Fluor™ 488	1:500	1% Donkey serum (TBS)	Donkey	Poly	Thermo Fisher	A21202; AB_141607
anti‐Rabbit IgG Alexa Fluor™ 594	1:500					A21207; AB_141637
anti‐Goat IgG Alexa Fluor™ 594	1:500					A11058;
Hoechst® 33342	1:2000	TBS	‐	‐	Thermo Fisher	H3570

^a^ Tris‐buffered saline.

### 2.6 High‐content imaging

PerkinElmer Opera Phenix High Content Screening System (RRID:SCR_021100) accompanied by Harmony version 4.1 (RRID:SCR_018809) were used to acquire and analyze images of cells after ICC. Images acquired on the Hoechst® 33342 channel were used to identify the nucleus of each cell (“Find Nuclei” module). Nuclei touching the border of each image were eliminated to ensure that a given nucleus is not counted more than once for downstream analysis (“Select Population” module). After calculating the “Area” and “Roundness” features of nuclei (“Calculate Morphology Properties” module), further filtering was applied based on these calculated features so that viable and nonclumped nuclei were chosen for downstream analysis (“Select Population” module). On this filtered population of nuclei, cytoplasmic region surrounding the nucleus for each cell was marked as a ring‐shaped region extending away from the nucleus (“Select Region” module, “Resize Region” method). The intensity features of this cytoplasmic region were calculated on images acquired from Alexa 488 and Alexa 568 channels (“Calculate Intensity Properties” module). Then, cells that met the threshold criteria for each channel intensity features were marked as being “positive” for a given cellular marker (“Select Population” module). The percentage of positive cells were reported at the end of analysis (“Define Results” module). Multiple negative‐control images were used (cells incubated only with secondary antibodies) to configure the correct threshold settings for each channel intensity. An example analysis pipeline is shown in Figure S1.

### 2.7 Statistics

All statistical analysis was performed on R Project for Statistical Computing v4.2.1 (https://cran.r-project.org/, RRID:SCR_001905) and RStudio Desktop 2022.07.0 + 548 (https://www.rstudio.com/, RRID:SCR_000432). Difference between the groups was evaluated with two‐way analysis of variance (ANOVA) followed by Bonferroni post hoc test for multiple comparisons. Mean ± standard deviation (*SD*), and the number of different cell passage numbers as replicates (*n*) are reported herein. Graphs were visualized with the ggplot2 (https://cran.r-project.org/web/packages/ggplot2/index.html, RRID:SCR_014601) and ggpubr (https://cran.r-project.org/package=ggpubr, RRID:SCR_021139) packages. The data used to generate the graphical figures and the multiple comparison test results are uploaded on Open Science Framework (osf.io/w67cd, RRID:SCR_003238).

### 2.8 Transcriptomics

#### 2.8.1 RNA‐sequencing and transcript abundance estimation

All cells for transcriptomics were cultured in six‐well plates. Total RNA was extracted using the RNeasy Mini Kit (QIAGEN, Cat# 74104) with on‐column DNase digestion (QIAGEN, Cat# 79254) according to the manufacturer's instructions. The cDNA library preparation prior to sequencing was performed with the poly‐A selection method to ensure that libraries were generated only from mRNA (NEBNext® Ultra™ RNA Library Prep Kit for Illumina®, New England BioLabs, Cat# E7530). Sequencing configuration was as follows: Illumina HiSeq 3000/HiSeq 4000 Systems (RRID:SCR_020127), 2 × 150 bp, single index, per lane. The number of reads per sample was 20–30 million (paired‐end), which is the standard sequencing setting suitable for differential gene expression analysis (Liu et al., 2014). Transcript abundance quantification on raw FASTQ files was carried out with the Salmon v0.14.0 (RRID:SCR_017036) (Patro et al., 2017) installed on the Ubuntu 18.04.3 LTS operating system. Pseudo‐alignment of the raw data files was performed using the index file generated for the GRCh38 human reference transcriptome downloaded from Ensembl (https://www.ensembl.org/Homo_sapiens/Info/Index, RRID:SCR_002344). Estimated abundance of each transcript was imported using the EnsDb.Hsapiens.v86 (Rainer et al., 2019) and the tximport (https://github.com/mikelove/tximport, RRID:SCR_016752) pipeline (Soneson et al., 2015), run on R Project for Statistical Computing v4.2.1 (https://cran.r-project.org/, RRID:SCR_001905) and RStudio Desktop 2022.07.0 + 548 (https://www.rstudio.com/, RRID:SCR_000432) installed on the Windows 10 Home operating system. The resulting output file contained gene level (rather than transcript level) summary of estimated counts.

Prior to exploratory analysis, the gene‐level estimated counts file was converted to a DESeq object using the DESeqDataSetFromTximport function embedded in the DESeq2 (https://bioconductor.org/packages/release/bioc/html/DESeq2.html, RRID:SCR_015687) package (Love et al., 2014). Filtering was applied on this object so that only the genes for which the estimated counts were more than or equal to 10 in all samples were kept for downstream analysis. For exploratory analysis, variance‐stabilizing transformation was performed on the counts file with the vst function (embedded in DESeq2) to make the range of variance similar across all samples (Anders & Huber, 2010). Then, principal component analysis and visualization of the top 35 most variable genes across all samples were performed on the variance‐stabilized counts. Raw data files and scripts used for analysis are uploaded on Open Science Framework (osf.io/w67cd, RRID:SCR_003238).

#### 2.8.2 Differential gene expression analysis

The *DESeq*() function of DESeq2 package was called on the nonvariance‐stabilized gene‐level estimated counts for differential gene expression analysis. Two groups were compared at a time using the Wald test. Shrinkage of effect size (log‐fold change values) was conducted prior to visualization of the results by calling the *lfcShrink*() function (type = “apeglm”) embedded in the DESeq2 package (Zhu et al., 2019). The cut‐off values for significance were false discovery rate (FDR)‐adjusted *p* values <10 E‐04 and log2 fold change values > |2|. Differentially expressed genes (DEGs) that were either unique to a given comparison or shared by two comparisons were also analyzed. Volcano plots were generated using the EnhancedVolcano (https://bioconductor.org/packages/release/bioc/html/EnhancedVolcano.html, RRID:SCR_018931) package (Blighe et al., 2022). Raw data files and scripts used for analysis are uploaded on Open Science Framework (osf.io/w67cd, RRID:SCR_003238).

#### 2.8.3 Gene set enrichment analysis

The *GSEA*() function in the clusterProfiler (http://bioconductor.org/packages/release/bioc/html/clusterProfiler.html, RRID:SCR_016884) package (Wu et al., 2021; Yu et al., 2012) was used to conduct gene set enrichment analysis against the Mammalian Adult Neurogenesis Gene Ontology (MANGO) (http://mango.adult-neurogenesis.de/, RRID:SCR_006176) database (Overall et al., 2012). DEGs of each timepoint (i.e., gene list) was matched against the “gene sets” curated in MANGO, which contains information on which “cellular stage” of HN a given gene expression is found to be “present” or “absent” in vivo (filtered by “Process/Outcome = Expression” AND “Effect = (+) OR (−)”). The DEG lists were sorted by log2 fold change values prior to analysis, and if a gene in the MANGO gene set (e.g., “Granule cell neuron”) was also found in the DEG list, the “running enrichment score” for that gene set was increased in proportion to the log2 fold change value of the gene. The results were visualized using the *gseaplot2*() function of the enrichplot package (Wu et al., 2021). Raw data files and scripts used for analysis are uploaded on Open Science Framework (osf.io/w67cd, RRID:SCR_003238).

#### 2.8.4 Gene ontology analysis

Enrichr (http://amp.pharm.mssm.edu/Enrichr/, RRID:SCR_001575) was used for gene ontology (GO) analysis (Chen et al., 2013; Kuleshov et al., 2016). Each list of DEGs was submitted to Enrichr, and the GO terms identified in the “Biological Process 2021” under the “Ontologies” section were examined. The “Table of top 10 significant *p* values and *q* values for GO Biological Process 2021” available on the Appyter view was downloaded as a csv file. The terms were sorted by −log10 *p* values (Fisher exact test) and visualized using the ggpubr (https://cran.r-project.org/package=ggpubr, RRID:SCR_021139) package. The csv files containing all GO terms identified for each DEG list, downloaded from the Appyter view of Enrichr, are uploaded on Open Science Framework (osf.io/w67cd, RRID:SCR_003238).

## 3 RESULTS

### 3.1 *APOE* genotype affects the expression pattern of *APOE* and neural progenitor genes relevant to hippocampal cell fate determination

**FIGURE 2:**
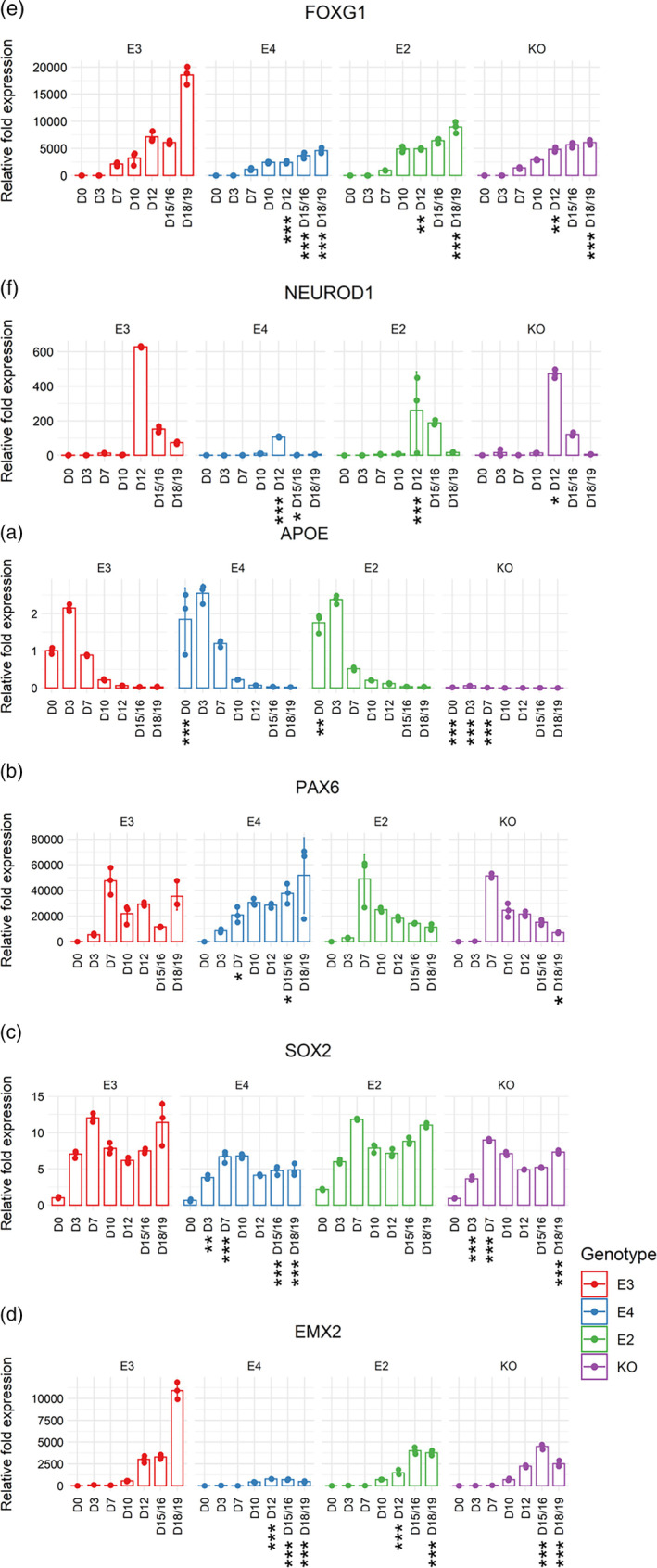
Gene expression patterns of APOE and neural progenitor genes crucial for determining hippocampal cell fate from Days 0 to 18/19 of directed differentiation. Real‐time qPCR was performed on isogenic *APOE* lines (E3, E4, E2, and KO). Expression values of (a) *APOE*, (b) *PAX6*, (c) *SOX2*, (d) *EMX2*, (e) *FOXG1*, and (f) *NEUROD1* were normalized to *GAPDH* expression. Day 0 of E3 was used as the reference sample for each gene. Two‐way ANOVA with Bonferroni correction. *n* = 3. Mean with *SD* shown. Adjusted *p* values: 0 < *** < 0.001 < ** < 0.01 < * < 0.05, when each cell line was compared to E3 on each day of directed differentiation.

To examine whether *APOE* expression is affected by its genotype during directed differentiation, qPCR was performed on *APOE* from Days 0 to 18/19 on isogenic *APOE* lines (Figure 2). We found that E4 and E2 iPSCs had a significantly higher expression of *APOE* compared to E3 on Day 0 (E4 adjusted *p* = .00011, E2 adjusted *p* = .00107), which is prior to directed differentiation (Figure 2a). However, *APOE* expression peaked at Day 3 and subsequently decreased in all cell lines, indicating no genotype‐specific effect.

We also compared the time course expression patterns of transcription factors expressed in NPCs that are known to be crucial for determining hippocampal cell fate, such as *PAX6*, *SOX2*, *EMX2*, *FOXG1*, and *NEUROD1* (Yu et al., 2014) from Days 0 to 18/19 (Figure 2b–f). In E3, E2, and KO cells, *PAX6* was most highly expressed on Day 7 and then gradually decreased until Day 18/19 (Figure 2b). Interestingly, E4 had a different expression pattern of *PAX6* compared to other genotypes: *PAX6* expression was significantly lower on Day 7 (vs. E3, adjusted *p* = .03711) and then gradually increased until Day 18/19. Despite the difference, the expression level of *PAX6* at Day 18/19 was not significantly different between E3 and other genotypes, except for KO which displayed a significantly lower expression.

In E3, E2, and KO cells, *SOX2* expression increased from Days 0 to 7 and then decreased until Day 12, followed by another increase until Day 18/19 (Figure 2c). While E2 and E3 were not significantly different at any timepoint observed in our study, E4 and KO cells had lower *SOX2* expression compared to E3 at several timepoints: E4 had lower expression on Days 3, 7, 15/16, and 18/19; while KO had lower expression on Days 3, 7, and 18/19. When comparing Days 10 and 7 expression within each cell line, we found that Day 10 expression was significantly lower than Day 7 in E3 (adjusted *p* = 2.0 e‐07) and E2 (adjusted *p* = 1.1 e‐06), while the level was similar between these two timepoints in E4 (adjusted *p* = 1) and KO cells (adjusted *p* = .59065), indicating the absence of a clear “peak” at Day 7.

*EMX2* (Figure 2d) and *FOXG1* (Figure 2e) expression increased steadily from Days 0 to 18/19 in all genotypes, but the “burst” of increase at Day 18/19 was evident only in E3 and was not observed in E4, E2, and KO cells. *EMX2* expression in E4 was significantly lower at Days 15/16 and 18/19 compared to other genotypes (at Day 15/16: E3 adjusted *p* = 1.2 e‐15, E2 adjusted *p* < 2 e‐16, KO adjusted *p* < 2 e‐16; at Day 18/19: E3 adjusted *p* < 2 e‐16, E2 adjusted *p* < 2 e‐16, KO adjusted *p* = 1.4 e‐11). FOXG1 expression in E4 was significantly lower across Days 12 and 18/19 compared to other genotypes, except for at Day 18/19 where it was similar with that of KO (adjusted *p* = .51957). Finally, *NEUROD1* expression peaked at Day 12 for all genotypes (Figure 2f), but notably, E4 had the lowest expression compared to other genotypes on this timepoint. Similarly, expression at Day 15/16 was also lower in E4 compared to other genotypes, except for KO (adjusted *p* = .44231).

Taken together, we report that *APOE* expression was significantly higher in E4 and E2 compared to E3 at the iPSC stage, and the time course expression of *PAX6* in E4 was different to that of other genotypes. *SOX2*, *EMX2*, *FOXG1*, and *NEUROD1* expression was significantly lower in E4 at varying timepoints between Days 3 and 18/19, while the overall time course expression pattern per se is comparable amongst the *APOE* genotypes.

### 3.2 Absence of *APOE* rather than genotype affects DGC‐like neuronal differentiation capacity

**FIGURE 3:**
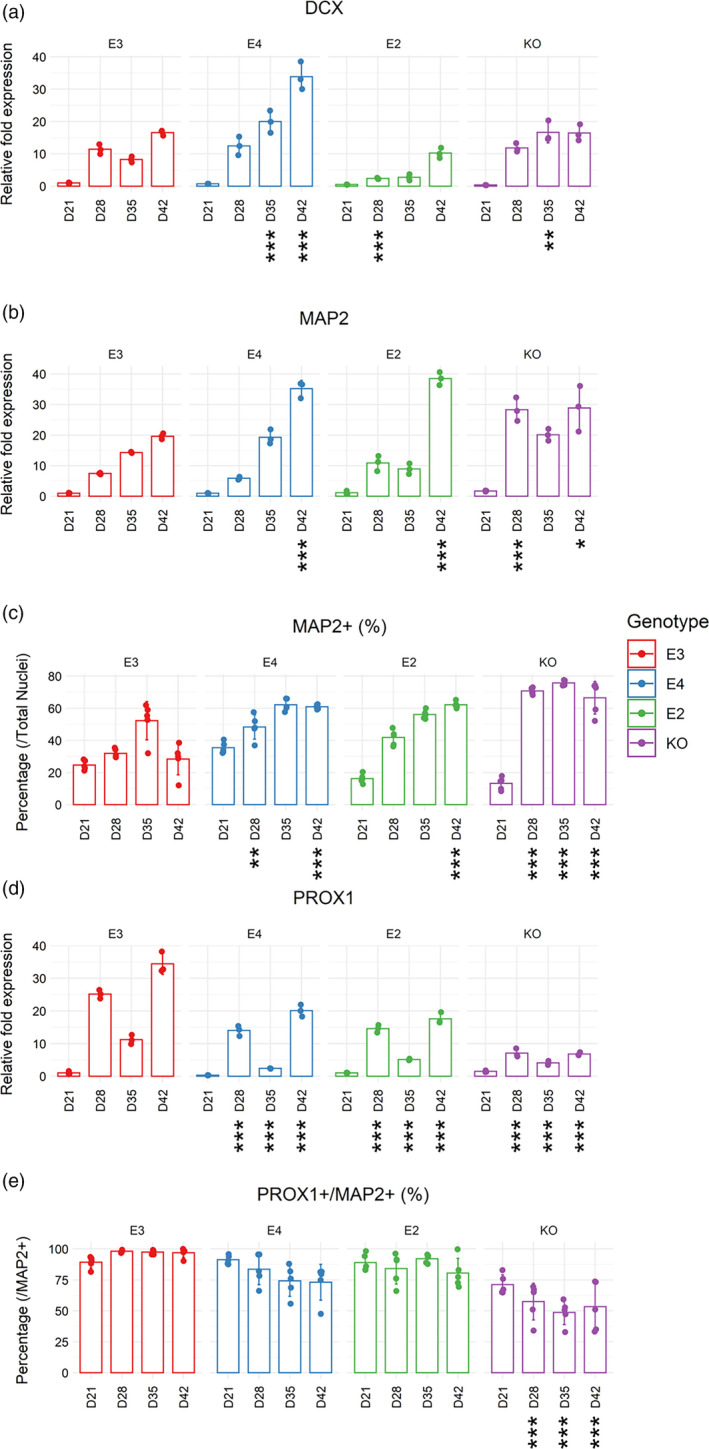
Characterization of DGC‐like neuronal differentiation capacity from Days 21 to 42 of directed differentiation. Real‐time qPCR was performed on isogenic *APOE* lines (E3, E4, E2, and KO). Gene expression of (a) *DCX*, (b) *MAP2*, and (d) *PROX1* were normalized to *GAPDH* expression. Day 0 of E3 was used as the reference sample for each gene. Two‐way ANOVA with Bonferroni correction. *n* = 3. Mean with *SD* shown. Immunocytochemistry for (c) MAP2+ cells (against total nuclei) and (e) PROX1+ cells (against MAP2+ cells) was performed on isogenic *APOE* lines (E3, E4, E2, and KO). Two‐way ANOVA with Bonferroni correction. *n* = 5. Mean with *SD* shown. Adjusted *p* values: 0 < *** < .001 < ** < .01 < * < .05, when each cell line was compared to E3 on each day of directed differentiation.

Next, we investigated whether the capacity to differentiate into DGC‐like neurons can be affected by *APOE* genotype. To this end, we measured the time course gene expression pattern of *DCX* (marker for neuroblasts), *MAP2* (marker for neurons), and *PROX1* (marker for DGC‐like neurons) from Days 21 to 42 of directed differentiation. Furthermore, we quantified % MAP2+ cells against the number of nuclei and % PROX1+ cells against % MAP2+ cells (denoted % PROX1+/MAP2+) during the same period via ICC, to measure the yield of “neurons in general” and “specifically DGC‐like neurons,” respectively (Figure 3).

**FIGURE 4:**
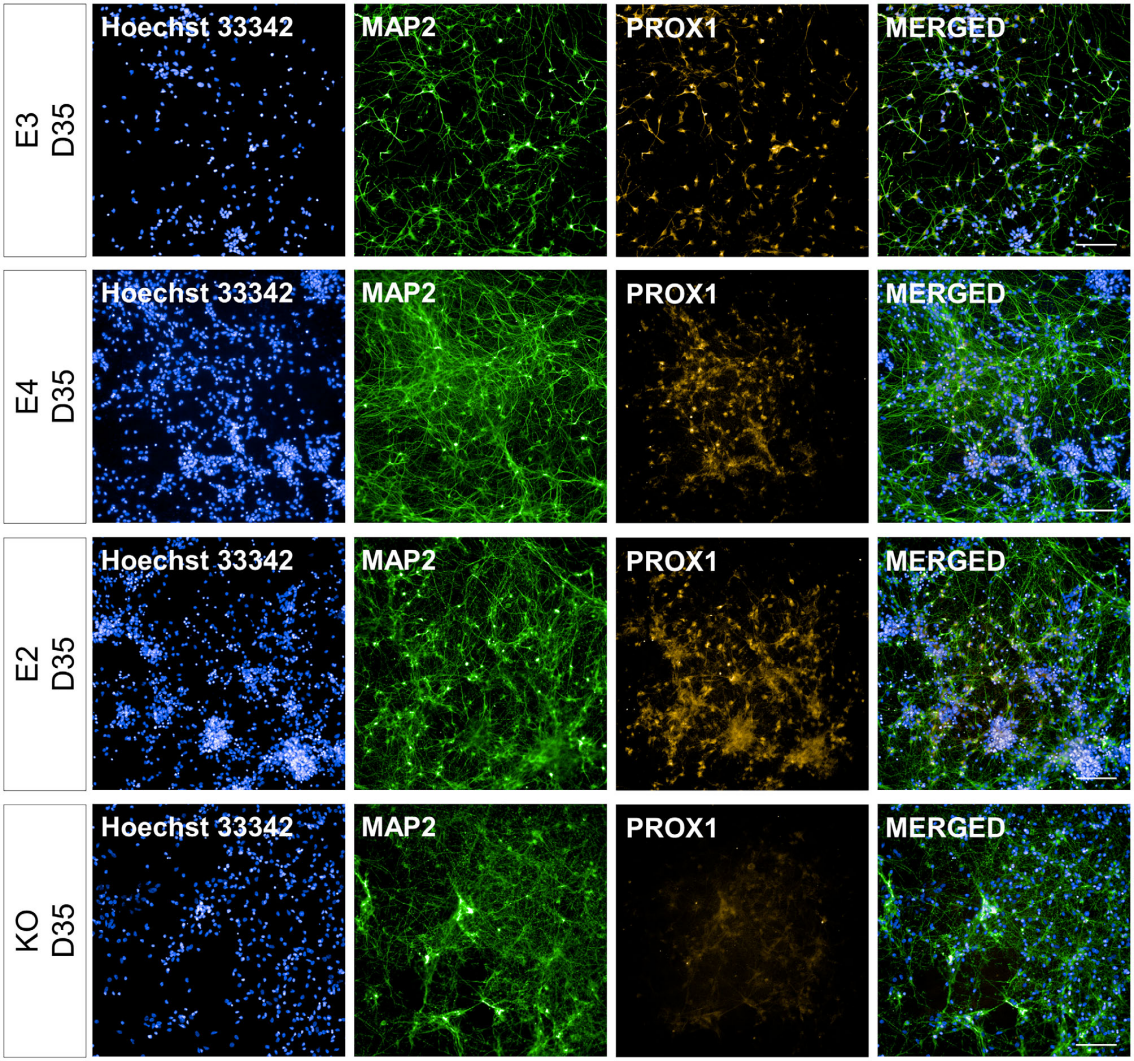
Representative images for MAP2+ and PROX1+/MAP2+ cells at Day 35 of directed differentiation. *APOE* isogenic lines (E3, E4, E2, and KO) were differentiated for 14 days from the date of terminal plating. MAP2 and PROX1 expression was evaluated via immunocytochemistry and high‐content imaging. Blue: Hoechst 33342.Green: MAP2. Orange: PROX1. Scale bar 100 μm

**FIGURE 5:**
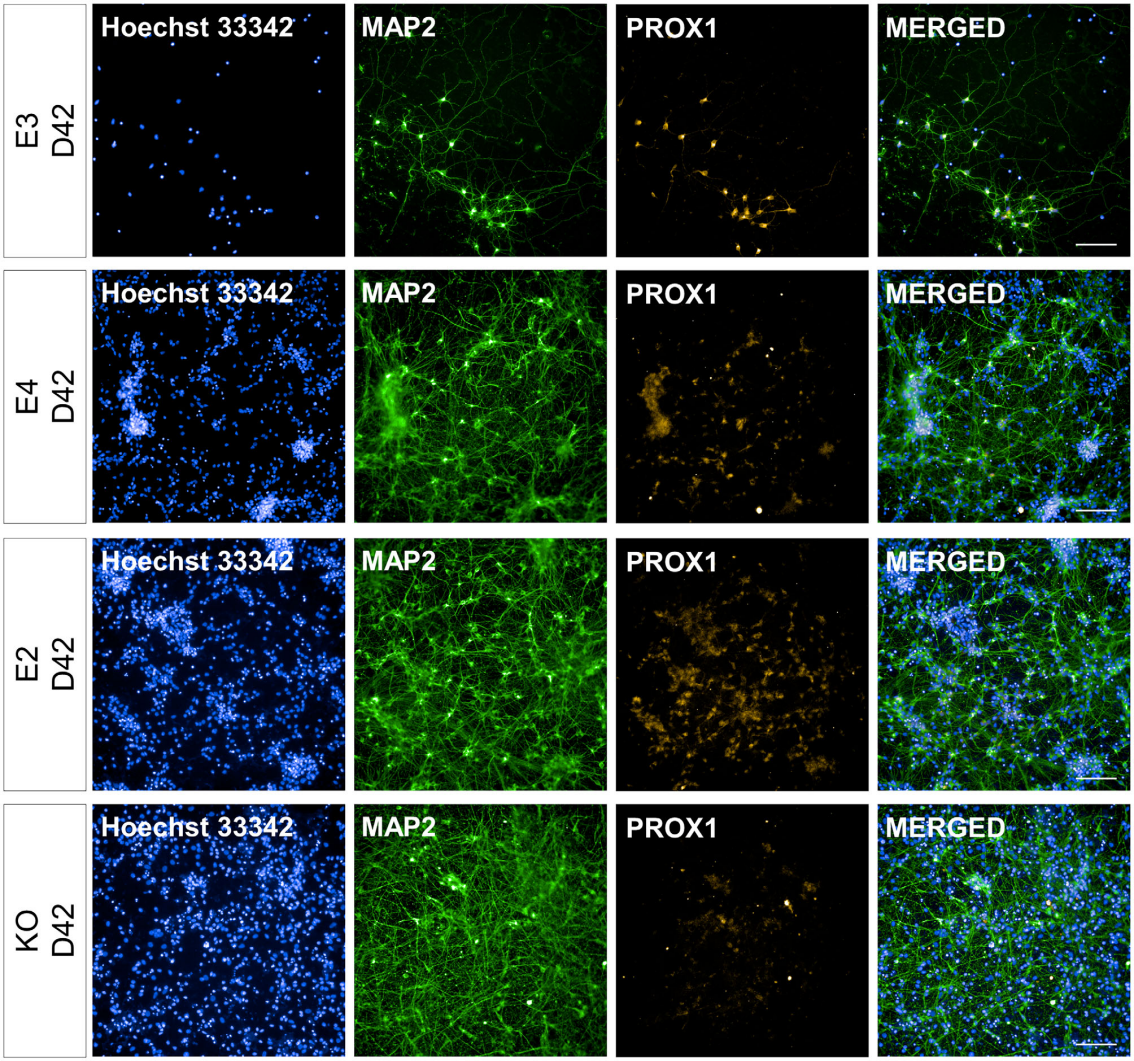
Representative images for MAP2+ and PROX1+/MAP2+ cells at Day 42 of directed differentiation. *APOE* isogenic lines (E3, E4, E2, and KO) were differentiated for 21 days from the date of terminal plating. MAP2 and PROX1 expression was evaluated via immunocytochemistry and high‐content imaging. Blue: Hoechst 33342.Green: MAP2. Orange: PROX1. Scale bar 100 μm

While *DCX* and *MAP2* gene expression showed a trend of increase (indicative of neurogenesis) in all genotypes (Figure 3a,b), E4 had a notably higher expression of *DCX* and *MAP2* gene as well as a lower expression of *PROX1* at Day 42 (Figure 3a,b,d). In line with this observation, ICC at Day 42 showed that E4 had 33% more MAP2+ cells compared to E3, while having 24% less PROX1+/MAP2+ cells. However, these differences between E4 and E3 were statistically significant only for MAP2+ cells (adjusted *p* = 1.7 e‐06) and not for PROX1+/MAP2+ cells (adjusted *p* = .06403) (Figure 3c,e). This suggests that the capacity to generate DGC‐like neurons is not severely compromised in E4 despite the lower gene expression level of *PROX1* (Figure 3d). In contrast, KO cells had higher % MAP2+ cells (38% more than E3, adjusted *p* = 6.9 e‐14) as well as significantly lower % PROX1+/MAP2+ cells (44% less than E3, adjusted *p* = 8.5 e‐07) compared to E3 at Day 42. Representative images of MAP2+ and PROX1+ cells are shown in Figures 4 and Figure 5.

**FIGURE 6:**
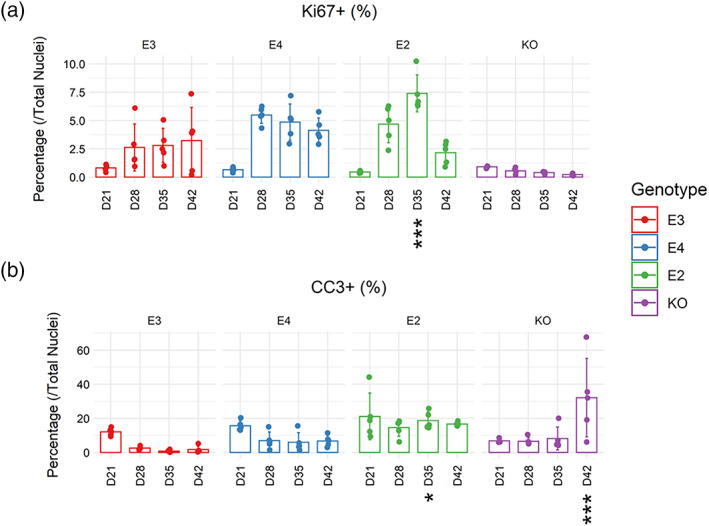
Characterization of proliferation and caspase‐mediated apoptosis from Days 21 to 42 of directed differentiation. Immunocytochemistry for (a) Ki67+ cells (proliferation) and (b) cleaved caspase 3 (CC3) + cells (apoptosis) was performed on *APOE* lines (E3, E4, E2, and KO). Both markers were normalized against the number of total nuclei. Two‐way ANOVA with Bonferroni correction. *n* = 5. Mean with *SD* shown. Adjusted *p* values: 0 < *** < .001 < ** < .01 < * < .05, when each cell line was compared to E3 on each day of directed differentiation.

**FIGURE 7:**
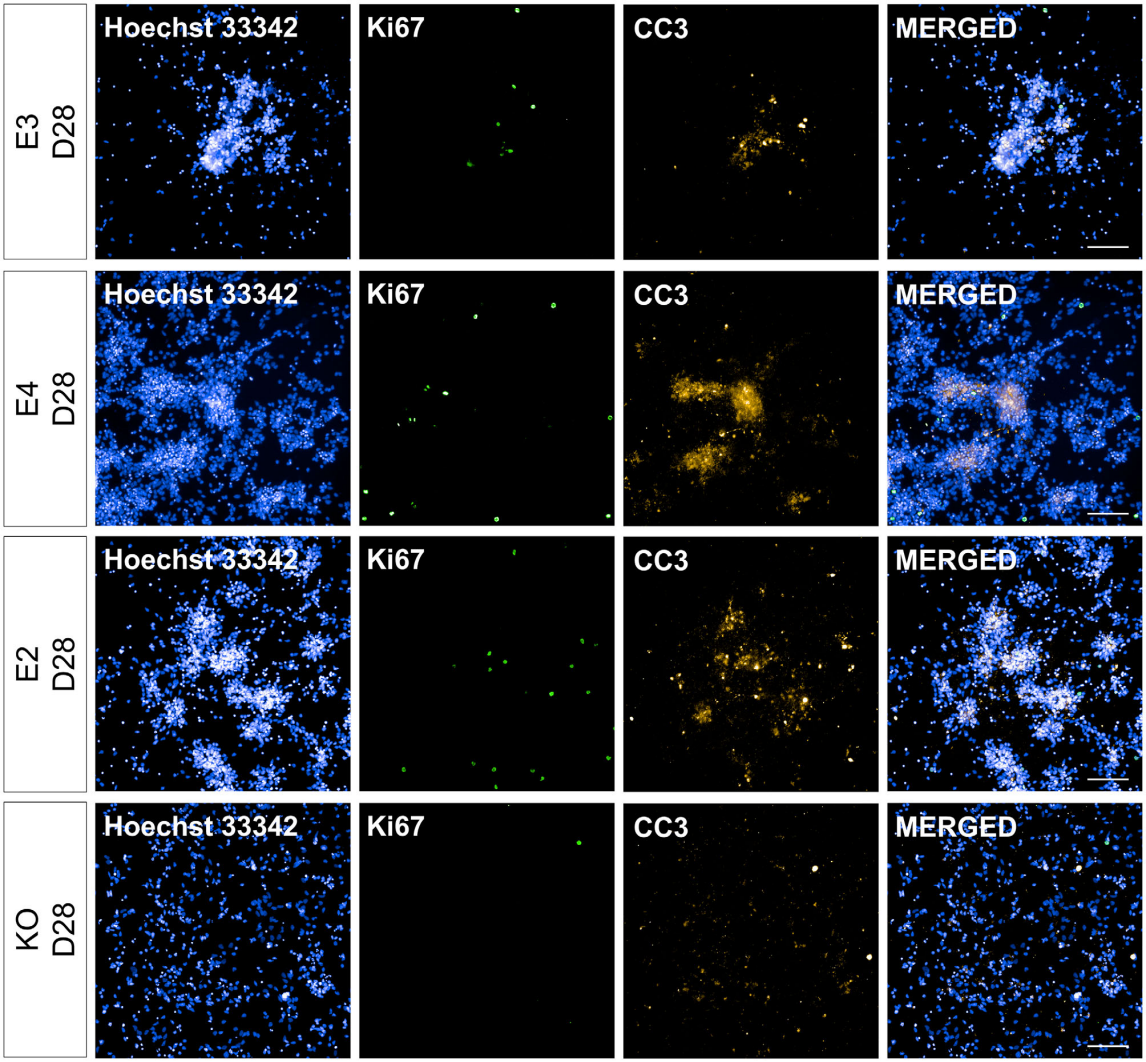
Representative images for Ki67+ and CC3+ cells at Day 28 of directed differentiation. *APOE* isogenic lines (E3, E4, E2, and KO) were differentiated for 7 days from the date of terminal plating. Ki67 and CC3 expression was evaluated via immunocytochemistry and high‐content imaging. Blue: Hoechst 33342.Green: Ki67. Orange: CC3. Scale bar 100 μm

We also examined the amount of proliferating and apoptotic cells during DGC‐like neuronal differentiation by quantifying the percentage of cells expressing Ki67 and cleaved caspase 3 (CC3), respectively (Figure 6). Both E3 and KO had little proliferation throughout neuronal differentiation (KO: 0.53 ± 0.29%, E3: 2.36 ± 2.03% across all timepoints), while E2 had higher levels of % Ki67+ cells compared to E3 at Day 35 (adjusted *p* = 4.0 e‐05). Moreover, all *APOE* genotypes had similar levels of % CC3+ cells throughout neuronal differentiation, while its level was higher in E2 and KO cells on Days 35 and 42, compared to E3, respectively (E2 at Day 35: 17.97% more vs. E3, adjusted *p* = .04297; KO at Day 42: 30.45% more vs. E3, adjusted *p* = 2.6 e‐06). Representative images of Ki67+ and CC3+ cells are shown in Figures 7 8.

**FIGURE 8:**
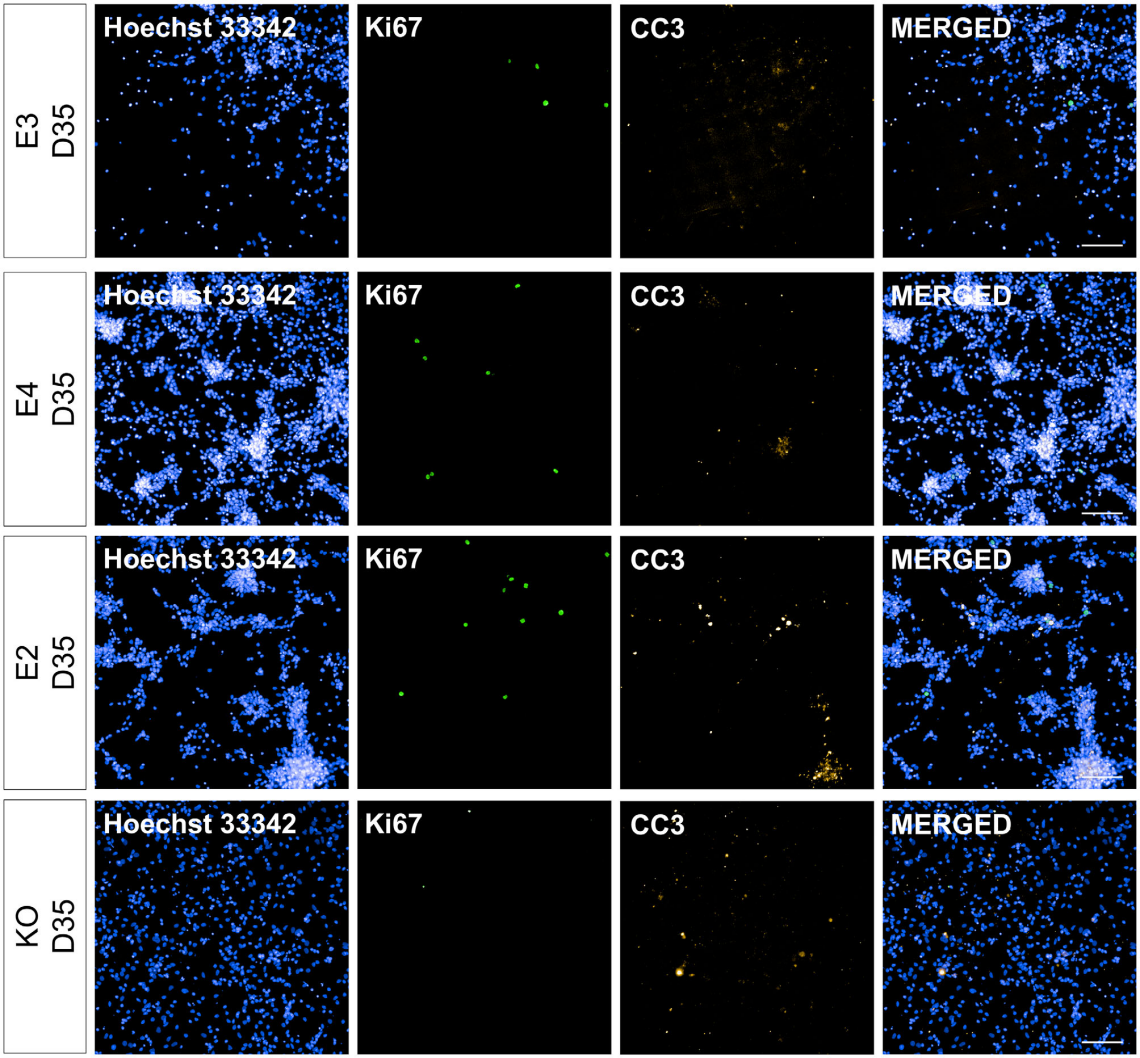
Representative images for Ki67+ and CC3+ cells at Day 35 of directed differentiation. *APOE* isogenic lines (E3, E4, E2, and KO) were differentiated for 14 days from the date of terminal plating. Ki67 and CC3 expression was evaluated via immunocytochemistry and high‐content imaging. Blue: Hoechst 33342.Green: Ki67. Orange: CC3. Scale bar 100 μm

Taken together, we show that *APOE* genotype can have differential impact on “neuronal differentiation in general’” (i.e., *MAP2* expression) and “DGC‐like neuronal differentiation” (i.e., *PROX1* expression), without significantly altering the yield of DGC‐like neurons in our model (i.e., % PROX1+/MAP2+ cells). Although % Ki67+ cells were higher in E2 at D35 compared to E3, all genotypes had similar levels of proliferation by D42. Moreover, E2 and KO cells were more apoptotic compared to E3 at D35 and D42, respectively. As shown by the significant reduction in PROX1+/MAP2+ cells in KO cells, DGC‐like neuronal differentiation capacity was affected more by the absence of *APOE* rather than different isoforms.

### 3.3 Exploratory analysis on transcriptomic data shows little evidence of *APOE* genotype‐dependent effect

The qPCR and ICC data described thus far suggest the following: (1) despite significant differences at certain stages of differentiation, the overall phenotypic difference observed in the *APOE* lines does not lead to impairment in DGC‐like neuronal differentiation capacity, (2) the gene expression phenotype characterized via qPCR could be more relevant to other cellular functions and pathways that were not investigated directly in our qPCR and ICC experiments, and finally, (3) *APOE* genotype may have little or no impact on the cells generated in our in vitro model of HN. To address these issues with a more sensitive method that would allow us to gain a more comprehensive understanding on the effects of *APOE* genotype, we performed a bulk RNA sequencing experiment on E3 and E4 cells at Days 3, 18, and 43 of directed differentiation. We aimed to investigate changes in gene expression phenotype in these cells at the “transcriptomic” level by conducting a series of differential gene expression analysis.

**FIGURE 9:**
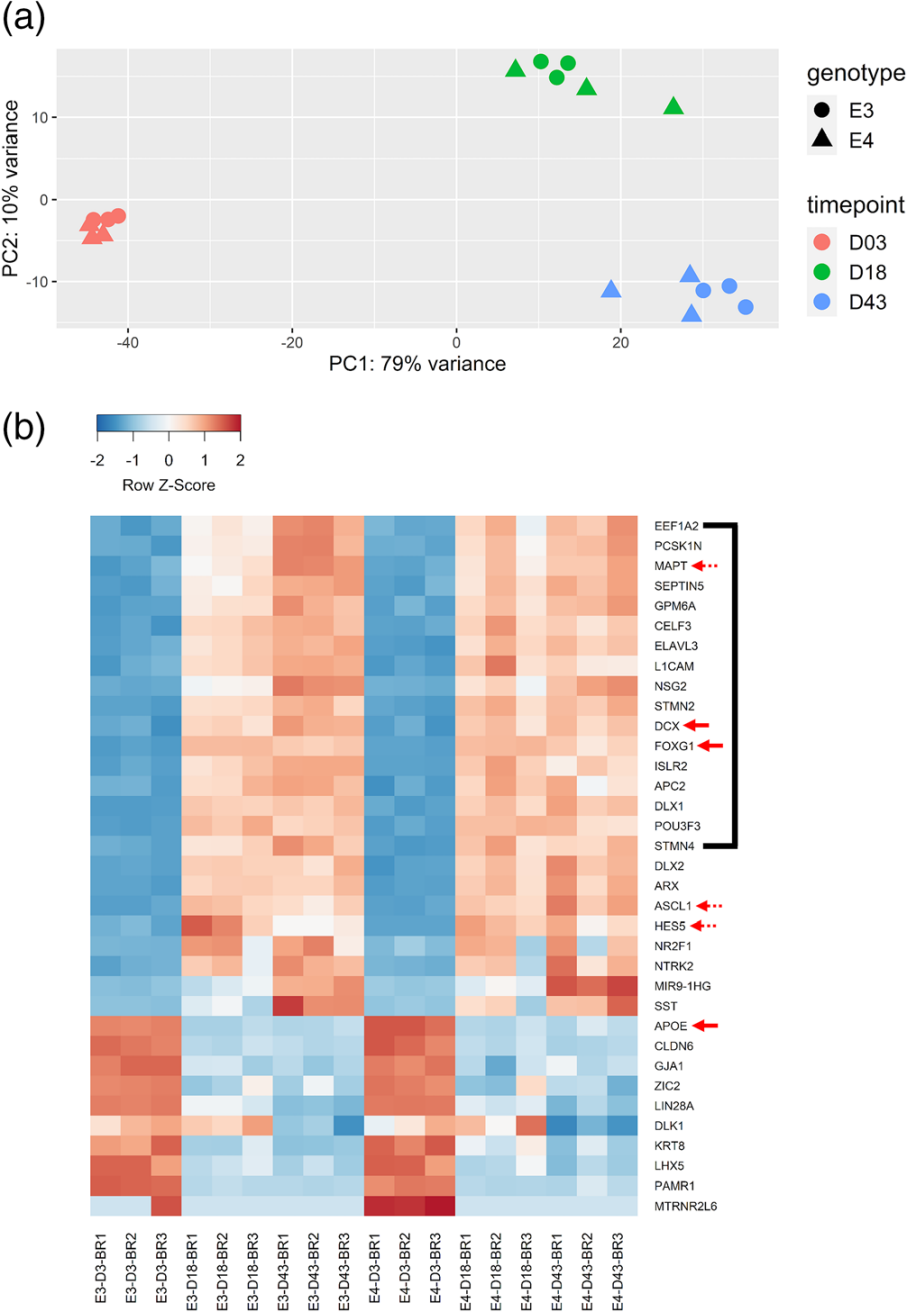
Principal component analysis on the transcriptome of E3 and E4 cells at Days 3, 18, and 43 of directed differentiation. (a) Principal component 1 (PC1) explains 79% of the variance across samples, and samples are clustered tightly according to different timepoints, regardless of genotype. Each data point indicates different cell passage number. (b) Heatmap of the top 35 most variably expressed genes across all samples. For genes listed from *EEF1A2* to *STMN4* on the right (black line), E3 cells show a more clear pattern of Day 3 < Day 18 < Day 43 transition compared to E4 cells. Red solid arrows indicate genes examined in the qPCR experiments (see Figure 2 for *APOE*, *FOXG1* and Figure 3 for *DCX*). Red dotted arrows indicate genes that were not examined in the qPCR experiments (*MAPT*, *ASCL1*, *HES5*), for which E4 cells show a less pronounced change in Z‐scores from Days 18 to 43, compared to E3. BR (replicate) indicates different cell passage number.

Principal component analysis based on the transcriptomic signature of the *APOE* lines confirmed that “timepoint” of directed differentiation (PC1: 79% variance explained) was the most significant factor of variance, and there was little indication of difference between E3 and E4 cells based on their transcriptomic signatures (Figure 9a). In line with this observation, the top 35 genes that varied the most across all samples distinguished the different “timepoints” of differentiation most clearly (Figure 9b).

The Z‐scores derived from the normalized expression matrix provided the following updates to our qPCR characterization. First, *DCX* expression was the highest at Day 43, but contrary to the qPCR data, the levels were comparable between E3 and E4. Second, *FOXG1* expression was confirmed to be the highest at Day 18, but contrary to the qPCR data, E3 and E4 had similar levels of expression at this timepoint, and it was Day 43 when the expression was down‐regulated in E4 compared to E3. Finally, *APOE* expression was confirmed to decrease with differentiation, and its level is also relatively higher in E4 at Day 3.

Exploratory analysis of the top 35 most variable genes also revealed changes in the following genes that were not directly characterized in our qPCR experiments. For instance, expression of *MAPT*, a gene up‐regulated in DCX‐positive neuroblasts (Llorens‐Martin et al., 2012), was increased in a linear fashion along differentiation in E3, while this trend was not very clear in E4. *ASCL1*, a pro‐neural transcription factor important for promoting neuronal differentiation in NPCs and neural stem cells (Andersen et al., 2014), was found to decrease in E3 and not as much in E4 at Day 43. Finally, a similar protracted expression was observed for *HES5*, another pro‐neural transcription factor highly expressed in neurogenic neural stem cells (Jin et al., 2003), in which the clear decrease from Days 18 to 43 was evident in E3 but not in E4.

### 3.4 Gene set enrichment analysis on differentially expressed genes confirm similar cellular identity for E3 and E4

To compare the cell fates assumed by E3 and E4 cells during directed differentiation, we conducted a gene set enrichment analysis (GSEA) on differentially expressed genes (DEGs) for the Day 18 versus Day 3 and Day 43 versus Day 18 comparisons for each genotype. The Mammalian Adult Neurogenesis Gene Ontology (MANGO) database (Overall et al., 2012) was utilized to determine which gene sets representing various “stages/cell types” of HN were enriched at Days 18 and 43 compared to their respective previous timepoints.

**FIGURE 10:**
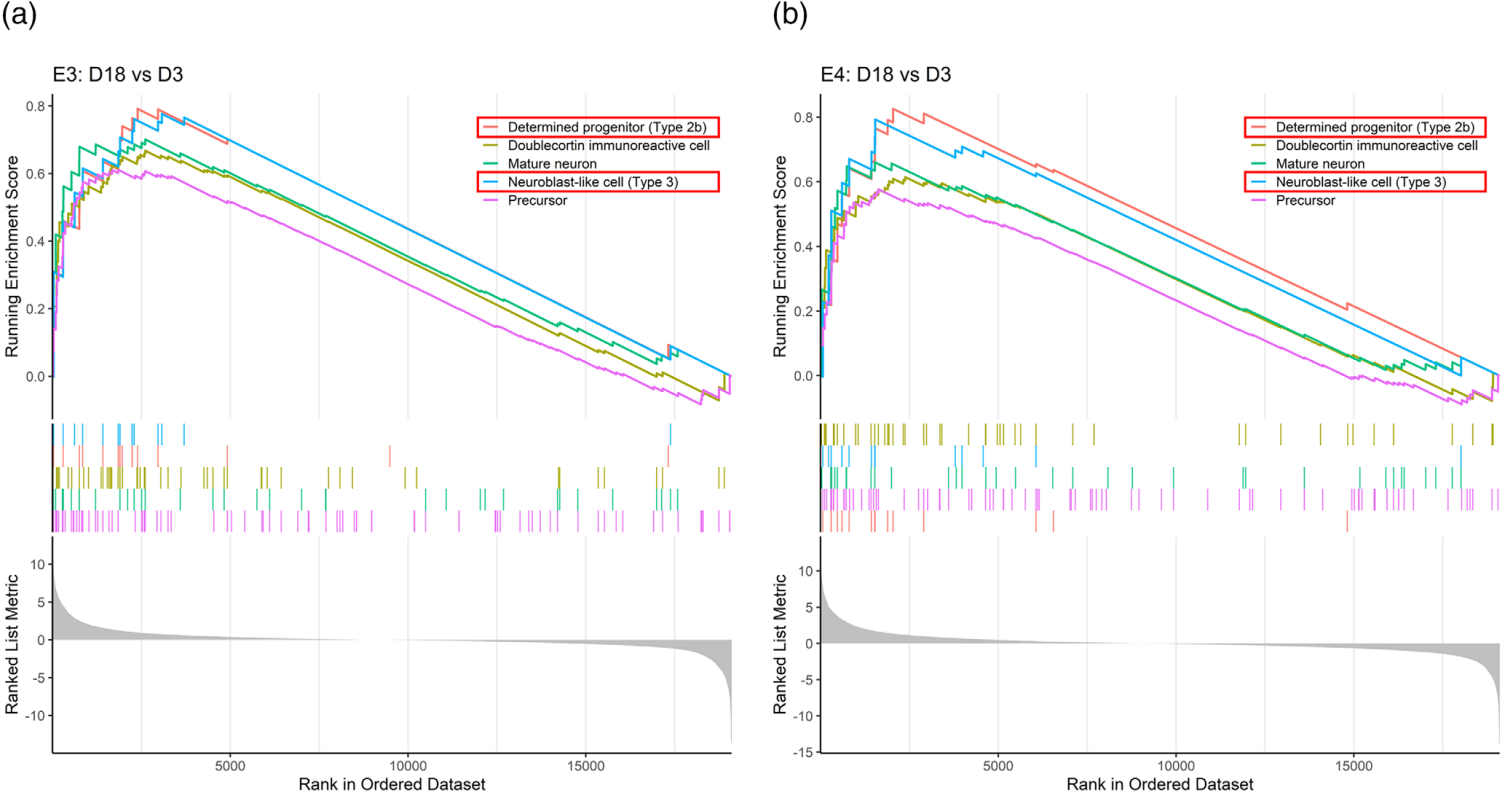
Gene set enrichment analysis shows similar identity of cells at Day 18 of directed differentiation for E3 and E4 cells. Differentially expressed genes (DEGs) at Day 18 versus Day 3 for (a) E3 and (b) E4 cells were sorted by log2 fold change values in decreasing order (i.e., gene lists). These gene lists were matched against the Mammalian Adult Neurogenesis Gene Ontology (MANGO) database gene sets. Genes with either “present (+)” or “absent (−)” effect on the outcome “expression” according to the MANGO database were used for enrichment analysis. Red box indicates the top two enriched gene sets in both E3 and E4 cells: “Determined progenitor (Type 2b)” and “Neuroblast‐like cell (Type 3).”

**FIGURE 11:**
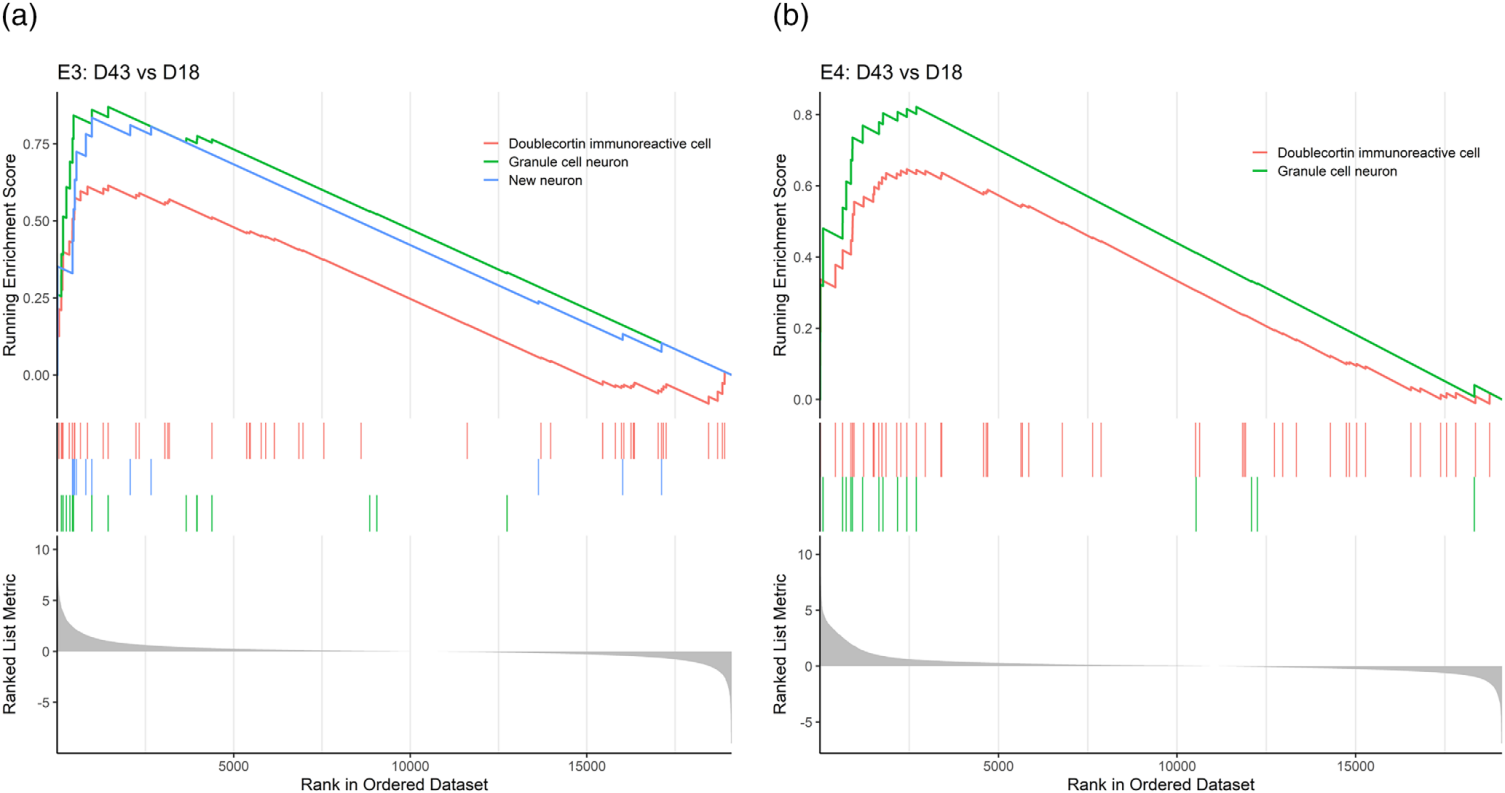
Gene set enrichment analysis shows similar identity of cells at Day 43 of directed differentiation for E3 and E4 cells. Differentially expressed genes (DEGs) at Day 18 versus Day 3 for (a) E3 and (b) E4 cells were sorted by log2 fold change values in decreasing order (i.e., gene lists). These gene lists were matched against the Mammalian Adult Neurogenesis Gene Ontology (MANGO) database gene sets. Genes with either “present (+)” or “absent (−)” effect on the outcome “expression” according to the MANGO database were used for enrichment analysis. Only two gene sets were identified to be enriched for E4 cells: “Doublecortin immunoreactive cell” and “Granule cell neuron.” The top enriched gene set in both E3 and E4 is “Granule cell neuron.”

We found that E3 and E4 had identical enriched gene sets at Day 18 compared to Day 3, in which the top two gene sets for both genotypes were “Determined progenitor (Type 2b)” and “Neuroblast‐like cell (Type 3),” confirming the NPC‐like state of both cell lines at Day 18 of directed differentiation (Figure 10). Similarly, at Day 43 compared to Day 18, the top two enriched gene sets for E3 were “Granule cell neuron” and “Doublecortin immunoreactive cell,” and they were the only gene sets identified to be enriched in E4, corroborating the DGC‐like neuronal state of both cell lines at Day 43 (Figure 11). Furthermore, Day 43 cells had high expression of genes such as *DCX*, stathmin 1 (*STMN1*), calbindin 2 (*CALB2*), neuronal differentiation 6 (*NEUROD6*), and roundabout guidance receptor 2 (*ROBO2*), all of which were shown to be highly expressed in human immature DGCs and neuroblasts expressing *PROX1* and *DCX* (Franjic et al., 2022; Zhou et al., 2022) (Figure S2 and Supplementary Discussion S1). Taken together, E3 and E4 cells are both likely to resemble the molecular signature of human immature DGCs at Day 43; and the GSEA against the MANGO database demonstrated that both E3 and E4 assume a similar cellular identity during directed differentiation (i.e., NPCs and DGC‐like neurons), despite significant differences in the time course expression pattern of transcription factors characterized via qPCR from Days 0 to 18 (Figure 2).

### 3.5 Phenotype divergence of E4 from E3 at Day 43 of in vitro HN

**FIGURE 12:**
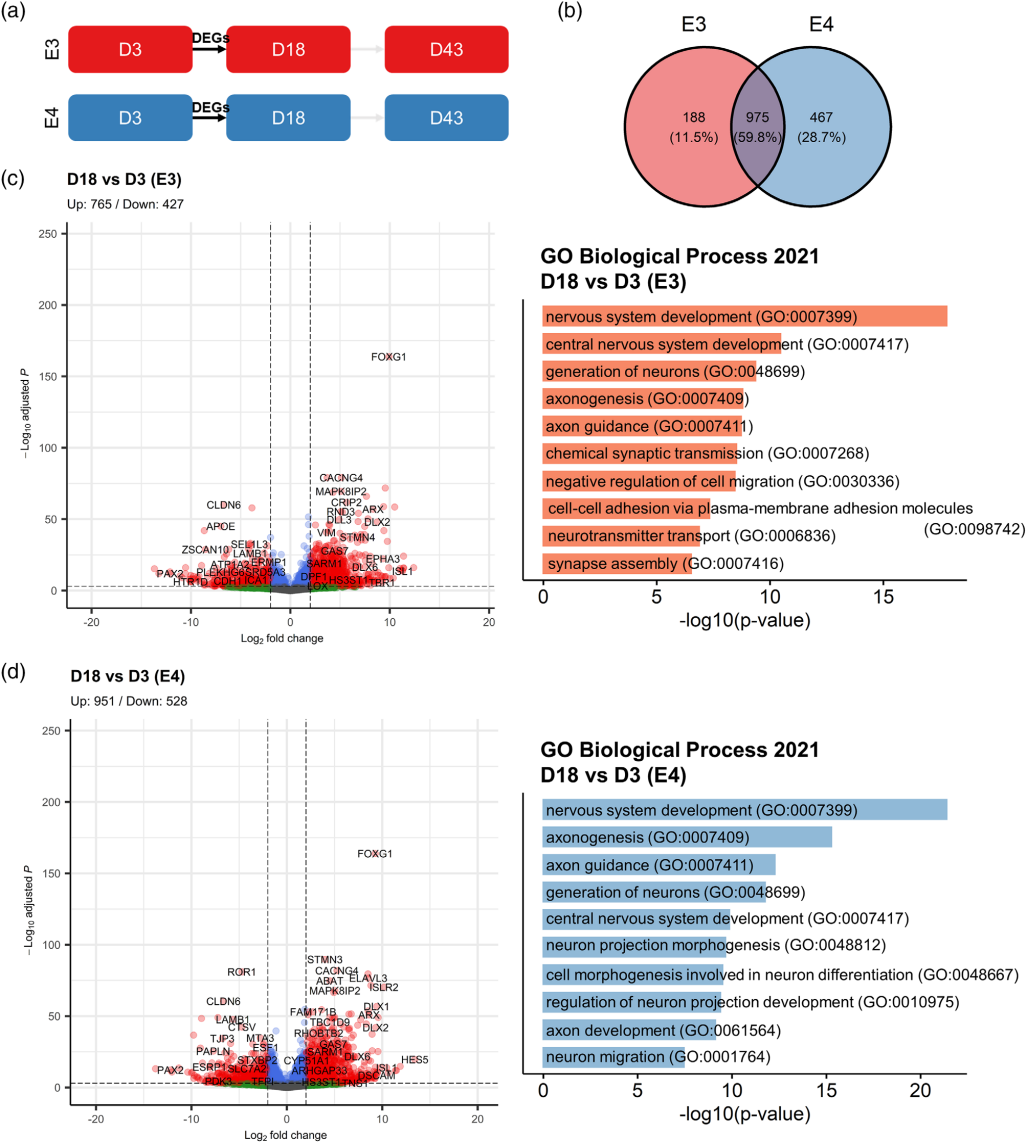
Differentially expressed genes at Day 18 of directed differentiation in E3 and E4 cells. (a) Schematic diagram of the analyzed DEGs. (b) Venn diagram showing the number (percentage) of unique and overlapping DEGs for E3 and E4 cells. DEGs in (c) E3 and (d) E4 are visualized in volcano plots (left), and the “Biological Process 2021” gene ontology (GO) terms identified for the DEGs (right) are shown. For the volcano plots, blue dots indicate Log2 fold change > |2|; green dots indicate −Log10 adjusted *p* values > 4; red dots indicate Log2 fold change > |2| and −Log10 adjusted *p* values > 4; and gray dots indicate nonsignificant. GO terms are sorted by −log10 *p* values.

**FIGURE 13:**
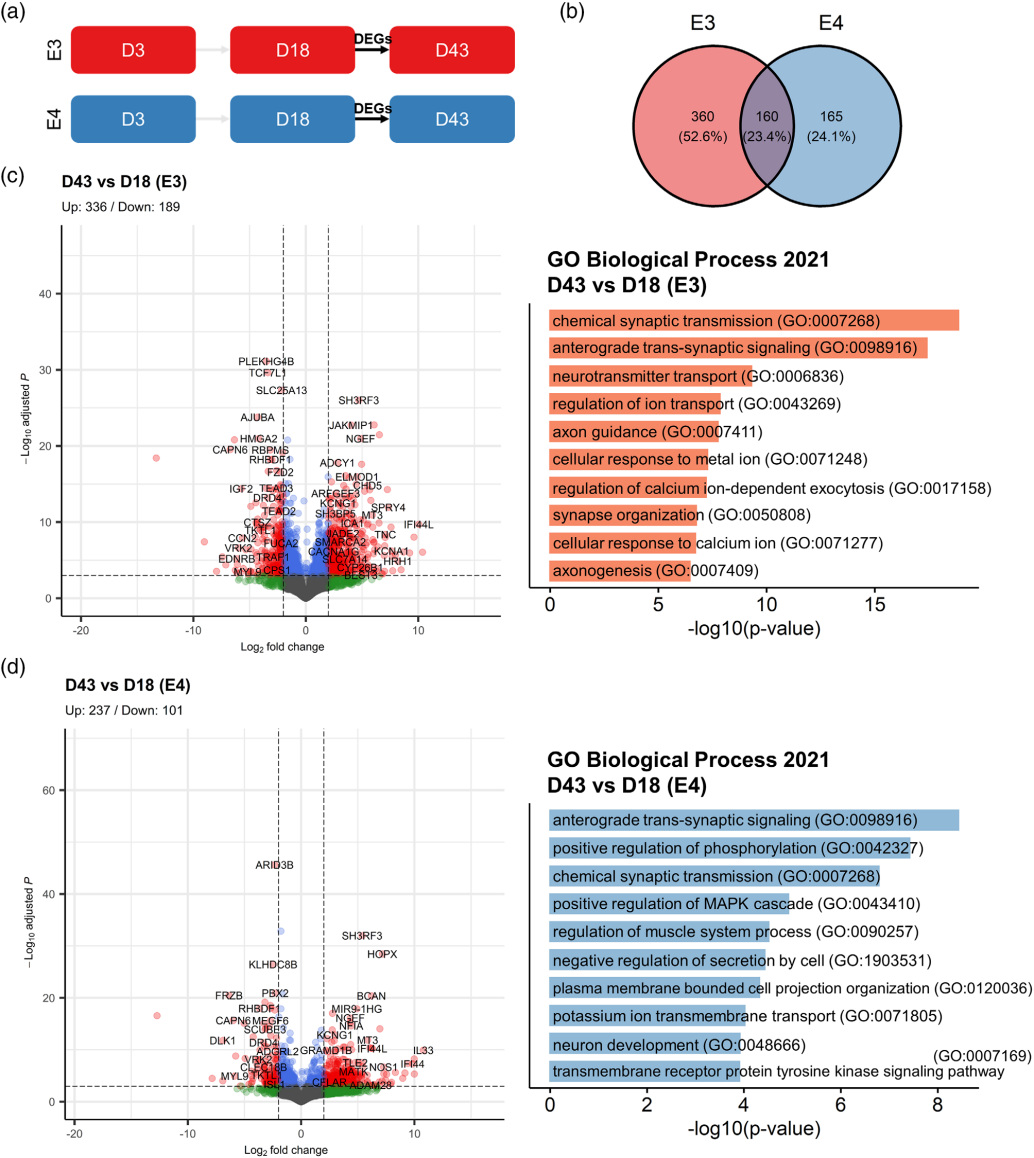
Differentially expressed genes (DEGs) at Day 43 of directed differentiation in E3 and E4 cells. (a) Schematic diagram of the analyzed DEGs. (b) Venn diagram showing the number (percentage) of unique and overlapping DEGs for E3 and E4 cells. DEGs in (c) E3 and (d) E4 are visualized in volcano plots (left), and the “Biological Process 2021” gene ontology (GO) terms for identified for the DEGs (right) are shown. For the volcano plots, blue dots indicate Log2 fold change > |2|; green dots indicate −Log10 adjusted *p* values > 4; red dots indicate Log2 fold change > |2| and −Log10 adjusted *p* values > 4; and gray dots indicate nonsignificant. GO terms are sorted by −log10 *p* values.

Having confirmed that cell fate determination is not significantly affected by *APOE* genotype in our model, we next asked whether the transcriptomic data could provide insight into cellular functions that could be altered by *APOE* isoforms. To this end, we analyzed the gene ontology (GO) terms for the DEGs of Day 18 versus Day 3 and Day 43 and Day 18 contrasts in each genotype. We found that the top 10 GO terms at Day 18 (Figure 12) were highly similar between E3 and E4, where “nervous system development (GO:0007399)” was the most enriched term for both genotypes (Figure 12c,d); and 59.8% of the DEGs found in E3 and E4 were shared between the two cell lines (Figure 12b). In contrast, at Day 43 (Figure 13), GO terms that were not as specifically relevant to neurogenic processes such as “regulation of muscle system process (GO:0090257)” was found in E4 (Figure 13c,d); and only 23.4% of the DEGs found in E3 and E4 were shared between the two genotypes (Figure 13b).

**FIGURE 14:**
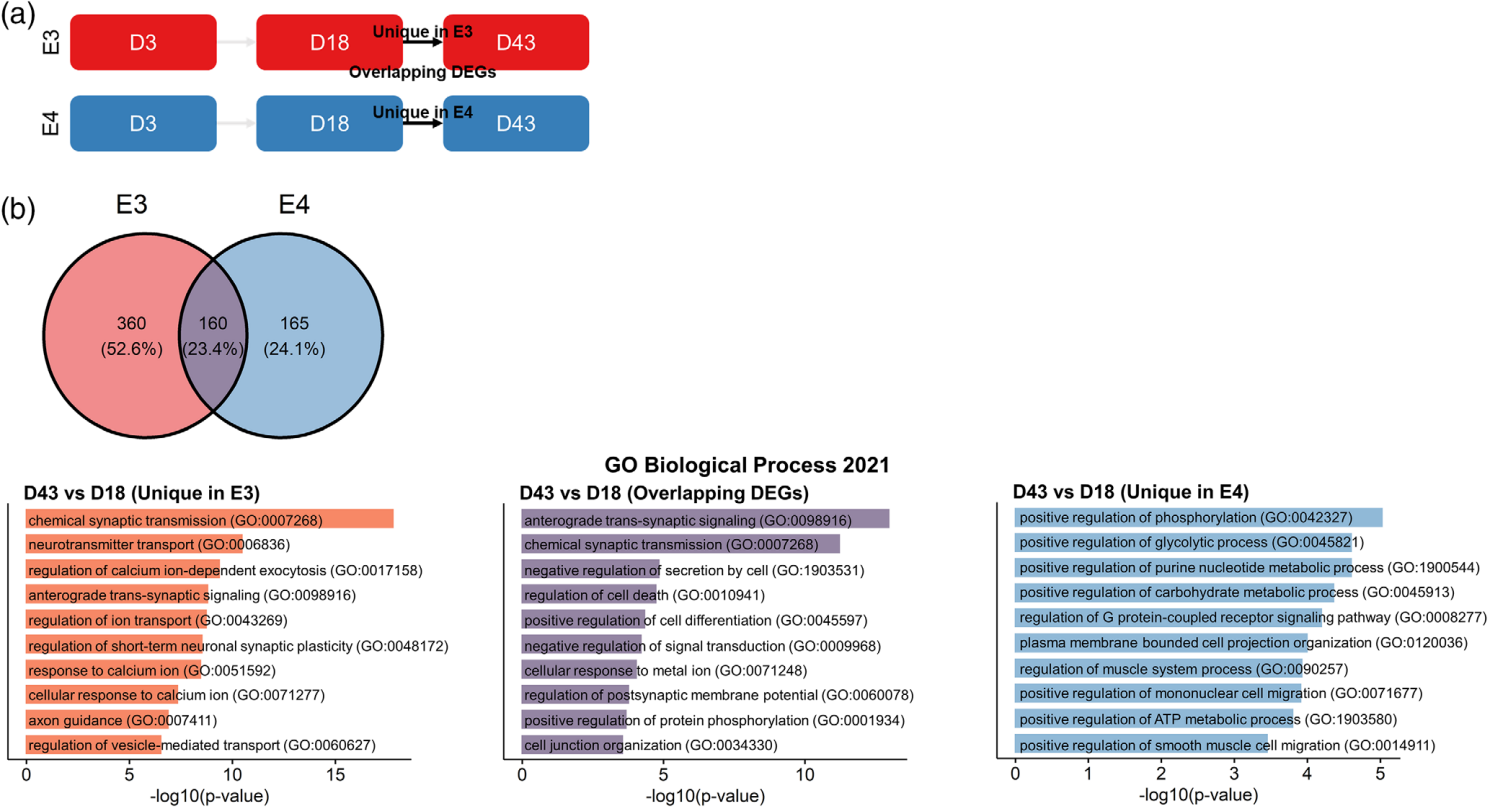
Differentially expressed genes at Day 43 of directed differentiation that are either unique to or shared by E3 and E4 cells. (a) Schematic diagram showing the differentially expressed genes (DEGs) analyzed. (b) Venn diagram showing the number (percentage) of unique and overlapping DEGs for E3 and E4 cells. The “Biological Process 2021” gene ontology (GO) terms for identified for the DEGs unique in E3 (bottom left), E4 (bottom right), and those shared by E3 and E4 (bottom middle) are shown. GO terms are sorted by −log10 *p* values.

**FIGURE 15:**
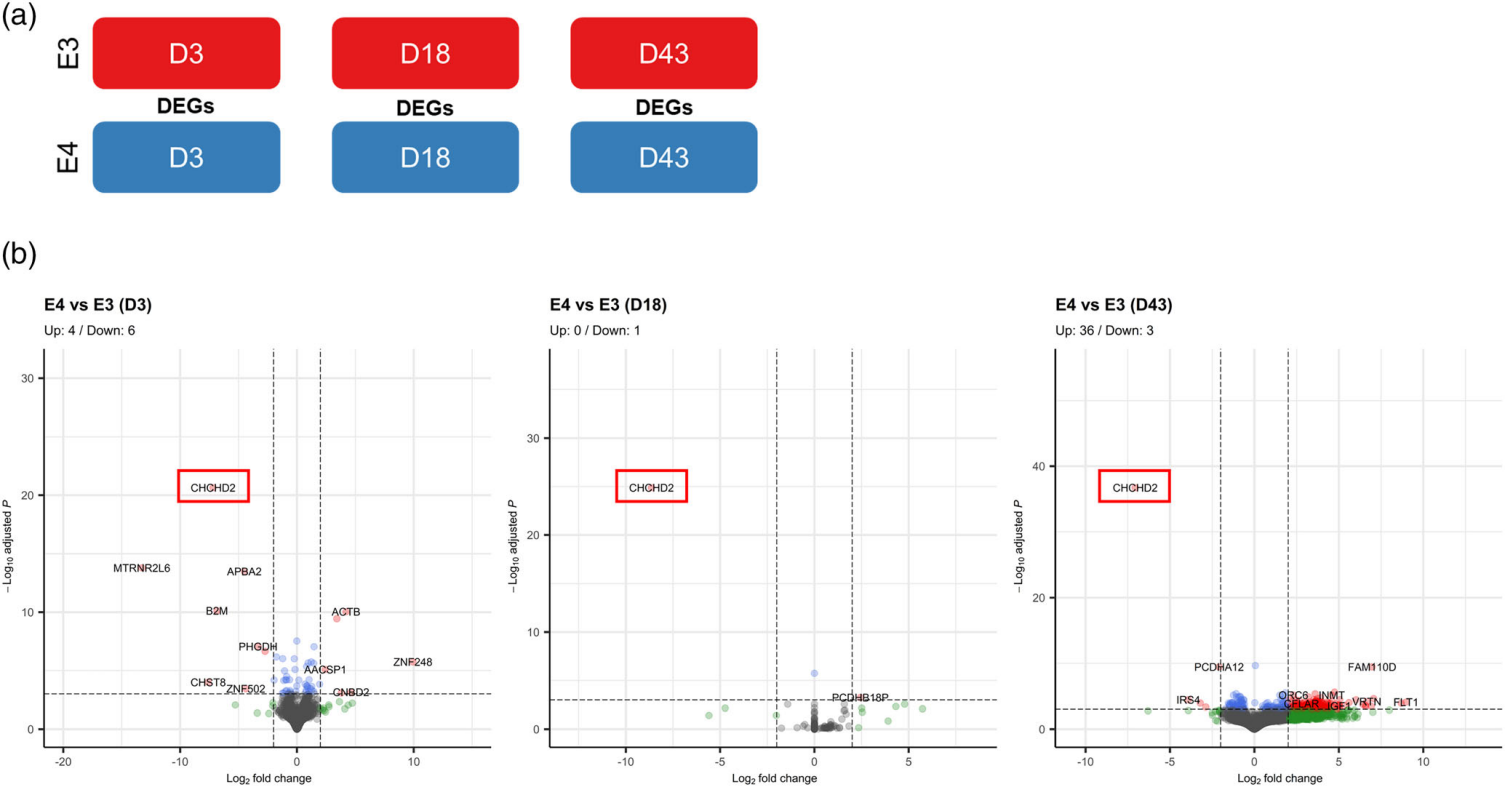
Differentially expressed genes in E4 compared to E3 at various stages of directed differentiation. (a) Schematic diagram showing the differentially expressed genes (DEGs) analyzed. (b) DEGs in E4 are visualized in volcano plots. Blue dots indicate Log2 fold change > |2|; green dots indicate −Log10 adjusted *p* values > 4; red dots indicate Log2 fold change > |2| and −Log10 adjusted *p* values > 4; and gray dots indicate nonsignificant. GO terms are sorted by −log10 *p* values. *CHCHD2*, a consistently down‐regulated gene in E4, is marked with a red box.

To further narrow down the GO analysis on functions that might be specifically altered in E4, we filtered the DEGs that were either unique to E3 or E4 for the Day 43 versus Day 18 comparison and conducted a separate GO analysis on these lists of DEGs. Strikingly, DEGs that were unique to E3 and DEGs that overlapped between E3 and E4 were clearly indicative of functional maturation of differentiated neurons (e.g., “chemical synaptic transmission (GO:0007268)” and “anterograde trans‐synaptic signalling (GO:0098916)”); while this was not the case for DEGs unique to E4 (Figure 14), where “positive regulation of phosphorylation (GO:0042327),” “positive regulation of glycolytic process (GO:0045821),” and “positive regulation of purine nucleotide metabolic process (GO:1900544)” were the top three enriched terms for E4‐only DEGs. When DEGs of E4 at each stage of directed differentiation were examined, we found that only 10, 1, and 39 genes were differentially expressed at Days 3, 18, and 43, respectively (Figure 15). Notably, one gene that was consistently found to be down‐regulated in E4 across all stages was coiled‐coil‐helix‐coiled‐coil‐helix domain containing 2 (*CHCHD2*). We also noted that the highest number of DEGs in E4 were identified at Day 43, confirming our observations on the phenotypic divergence at this timepoint shown in Figures 13 and 14. Taken together, the transcriptomic data suggest that E3 phenotype more clearly indicates “functional maturation” of neurons, while E4 phenotype diverges at this stage (Day 43) from that of E3, hinting at potential alterations in various metabolic processes and most prominently characterized by consistent downregulation of *CHCHD2*.

## 4 DISCUSSION

In this study, *APOE* genotype‐dependent phenotypes of HN were characterized in vitro using isogenic human iPSCs. To develop a better understanding on the time‐dependent changes during directed differentiation, we took a time‐course characterization approach in this study and found that (1) *APOE* is highly expressed at earliest stages of directed differentiation, (2) *APOE* genotype affects the gene expression pattern of transcription factors critical for hippocampal cell fate determination without altering the DGC‐like neuronal differentiation capacity, and finally, and (3) the phenotype divergence of E4 from E3 occurs more prominently during the neuronal differentiation stage of our model.

We have previously shown that *APOE* expression decreases drastically as the cells become more differentiated from NSCs into NPCs of nonhippocampal lineage in vitro (Lee et al., 2020), and we report here a similar finding in our model of HN. These observations collectively suggest a role of *APOE* in stem cell maintenance/differentiation, in line with other independent studies on mice (GEO accession: GSE22908) (Polo et al., 2010), and humans (GEO accession: GSE20750) (Nishino et al., 2011; Saito et al., 2011; Tateno et al., 2011), where *APOE* was consistently found to be expressed more highly in “less” differentiated cells. With regards to genotype‐dependent differences, here we show that *APOE* is more highly expressed in E4 and E2 compared to E3 at the iPSC stage, prior to directed differentiation, although the exact “functional significance” of these differences continues to remain an avenue for further research.

Since the absence of *APOE* in stem cells resulted in lower yield of PROX1+/MAP2+ DGC‐like neurons upon neuronal differentiation, substantial expression of *APOE* at the stem cell stage seems to be critical for HN. However, this is not likely to be attributed to alterations in transcription factor expression at the NPC stage, because knockout had little effect on the temporal dynamics of their expression prior to neuronal differentiation. Even if significantly lower expression of some genes at certain timepoints are taken into consideration, it should be noted that E4, for which most transcription factor expression was significantly lower than E3 at many timepoints, had little alterations in the DGC‐like neuronal differentiation capacity at Day 42/43. While notable difference was observed in *PAX6* temporal expression in E4, which might have directly contributed to lower expression of *EMX2* and *NEUROD1* after Day 7 (Sansom et al., 2009), the effect of E4 genotype does not seem to extend beyond Day 18/19 of directed differentiation, because the yield of PROX1+/MAP2+ DGC‐like neurons was ultimately not affected. We do note that higher *MAP2* and lower *PROX1* gene expression was observed after Day 18/19 in E4 cells, but this was also the case in E2 and KO; the former having similar yield of DGC‐like neurons as E3, while the latter producing significantly lower percentage of this population. The low yield of PROX1+/MAP2+ cells in KO could be related to enhanced caspase‐mediated apoptosis, which was also a phenotype we observed only in KO, but further investigation would be required to examine the hypothesis of PROX1+ cells of MAP2+ population being more susceptible to programmed cell death.

While it seems clear that the presence or absence of *APOE* is a more deterministic factor of DGC‐like neuronal differentiation capacity, the E4 genotype did correlate with the divergence of phenotype from that of E3 during the neuronal differentiation period at the transcriptomic level. This supports previous findings of E4 cells having more substantial changes in their transcriptome at the neuronal/glial differentiation stage (Lin et al., 2018). GO terms identified prior to Day 43 were highly relevant to neuronal maturation in both E3 and E4 cells, which included axonogenesis and chemical synaptic transmission, indicating that both cell lines had comparable capacity to generate mature neurons poised to become electrochemically active. GSEA also showed that both E3 and E4 assumed the DGC identity at Day 43, confirming the qPCR and ICC characterization data.

However, when “Day 43 versus Day 18” profiles of E4 and E3 cells were segregated into “E3‐only,” “overlapping between E3 and E4,” and “E4‐only” DEGs, we found that GO terms for E4‐only DEGs were enriched with genes highly expressed during phosphorylation, glycolysis, and purine nucleotide metabolism. Combined with the decreased expression of *CHCHD2* we observed at all three timepoints of RNA‐seq, our data suggest that the phenotypes distinguishing E4 from E3 are likely to be related to alterations in metabolism and mitochondrial function. *CHCHD2* has been previously shown to localize at the intermembrane space of mitochondria (Aras et al., 2015; Funayama et al., 2015), and decreased expression and/or loss has been associated with mitochondrial dysfunction (Funayama et al., 2017). Long‐term damage in (mitochondrial) metabolism can detrimentally affect brain regions like the DG, where energy demands are high due to ongoing neurogenesis, even though the cells are equipped with the correct molecular machinery to generate DGCs. Interestingly, altered metabolism linked to changes in brain function is one of the most robust phenomena reported consistently in various observational studies of ε4 carriers (Liu et al., 2015). However, it should be noted that this study did not examine the functional differences of E4 and E3 cells, and further experiments would be necessary to validate the phenotypic inferences one can make from our transcriptomic dataset. Therefore, we propose that follow‐up studies should focus on elucidating the relationship between altered (mitochondrial) metabolism and *APOE* genotype in the context of HN.

In addition, we recognize the following limitations of this study. The isogenic cell lines contained only one functional allele of *APOE* despite expressing the correct isoform (Schmid et al., 2020) (except KO, which had no functional *APOE* expressed altogether). Although the expression of both alleles in each cell line would have either provided “more” or “better” insight into the differences between *APOE* genotypes, our data alone suggest that SNPs on a “single” allele can exert transcriptome‐wide effects that clearly resonate with other independent studies that found “altered metabolism and mitochondrial function” as a potential mechanism of E4 under homozygous conditions. Another limitation is that our model does not distinguish embryonic and adult HN. The existing literature on *APOE* genotype and HN hints at adult neural stem cell pool exhaustion (Yang et al., 2011) and maturation failure of adult‐born neurons (Li et al., 2009) in the DG as important phenotypes in rodents expressing either no *APOE* or human ε4, respectively. Both neural stem cells and newborn neurons in the “adult” DG have been shown to bear significant functional differences from their embryonic counterparts (Cole et al., 2020; Urbán & Guillemot, 2014). Therefore, a more specific modeling of “adult” HN could be yield further insights that would have been difficult to obtain using our model. Furthermore, it is important to note that in vitro models of HN, such as the one used in this study, cannot fully recapitulate the complexity of the in vivo DG niche. The DG is known to be highly vascularized (Shen et al., 2019) and consists of many types of cells, such as inhibitory neurons, glial cells, oligodendrocytes, and endothelial cells, all of which have a significant influence on the course and outcomes of HN (Li et al., 2009; Morrens et al., 2012; Seki, 2003). A more “in vivo‐like” model of HN that takes many of these cell types into account would be especially relevant for the investigation of *APOE*, since it is known to be highly expressed in glial cells such as astrocytes and microglia (Liu et al., 2013). Nevertheless, the simplicity of the model allowed us to isolate the “cell‐autonomous” effects of *APOE* in HN, and we show here that *APOE* expression in neural stem cells, in and of itself, is crucial for HN.

In summary, our in vitro model of human HN and isogenic *APOE* lines demonstrate that *APOE* is differentially expressed at the stem cell stage, while the phenotypic divergence of E4 from E3 became more prominent at the neuronal stage of differentiation, without substantial changes to DGC‐like neuronal differentiation. Genes associated with “maturation of functional neurons” were more clearly expressed in E3 neurons compared to E4, while genes uniquely expressed in E4 neurons indicated alterations in metabolism and mitochondrial function. Future investigations on these potential “vulnerabilities” could provide better insight into the mechanistic link between defects in metabolism and hippocampal function, both of which are frequently observed in ε4 carriers.

## AUTHOR CONTRIBUTIONS

**Hyunah Lee**: Conceptualization, Data Curation, Formal Analysis, Investigation, Methodology, Visualization, Writing, Review & Editing. **Jack Price**: Conceptualization, Resources, Review & Editing. **Deepak P. Srivastava**: Conceptualization, Review & Editing. **Sandrine Thuret**: Conceptualization, Funding Acquisition, Project Administration, Resources, Supervision, Writing, Review & Editing.

## CONFLICT OF INTEREST STATEMENT

The authors declare no competing interest.

## Supporting information


**Data S1.** Supporting Information.

## Data Availability

The data that support the findings of this study are openly available at Open Science Framework (osf.io/w67cd).
