# Supplementary material for: In vitro characterization on the role of APOE polymorphism in human hippocampal neurogenesis
Source: Hippocampus. 2023 Jan 28;33(4):322–46. doi: 10.1002/hipo.23502 (PMC10947111; doi:10.1002/hipo.23502)
Supplement: Supplementary file 1 — Data S1. Supporting Information. [file HIPO-33-322-s001.docx]

# Supporting Information

**Supplementary Figure 1 An example analysis pipeline for high-content imaging with Opera Phenix.**

Harmony software 4.1 (Perkin Elmer) was used to select the modules for a given input (e.g. image or a population) (left column) that used a specific method (middle column) to calculate the ouput population (right column). At the “Define Results” module, the percentage population normalised to a given reference population (e.g. nuclei, MAP2+ cells) was calculated.


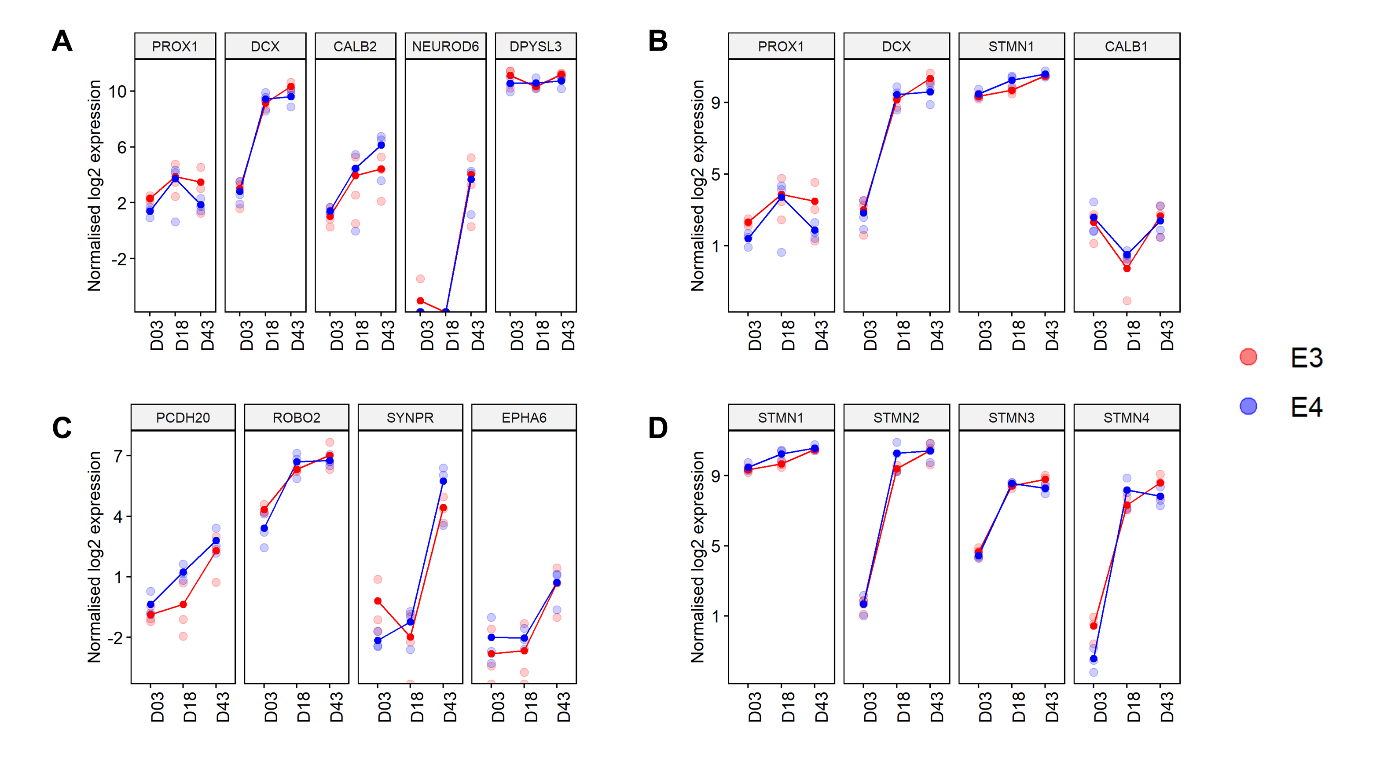


**Supplementary Figure 2 Expression of genes enriched in human immature and mature DGCs from published single-nucleus RNA-seq studies examined in E3 and E4 cells**

Normalised log2 expression of genes enriched in A) human neuroblasts expressing *PROX1* and *DCX* according to the Franjic et al. 2022 (*Neuron*) study; B) human immature DGCs (i.e., *PROX1, DCX, STMN1*) and mature DGCs (i.e., *CALB1*) in the Zhou et al. 2022 (*Nature*) study; and C) human immature DGCs (i.e., *PCDH20, ROBO2*) and mature DGCs (i.e., *SYNPR, EPHA6*) in the Zhou et al. 2022 (*Nature*) study. D) Expression of Stathmin family genes (i.e., *STMN2, STMN3, STMN4*) alongside *STMN1* in our transcriptomic dataset. Hollow dots indicate different passage number replicates of each cell line (red = E3, blue = E4). Solid dots indicate the mean of replicates (red = E3, blue = E4). R scripts supporting these graphs are openly available at Open Science Framework (osf.io/w67cd).

## Supplementary Discussion

We compared our bulk RNA-seq dataset to recently published single nucleus RNA-seq (snRNA-seq) datasets (Franjic et al., 2022; Zhou et al., 2022) to examine the temporal expression pattern of genes that were previously shown to be enriched in immature and mature DGCs in the human brain. We first looked at genes that were highly expressed in human DG neuroblasts according to Franjic and colleagues’ dataset, such as *PROX1*, *DCX*, *CALB2*, *NEUROD6*, and Dihydropyrimidinase Like 3 (*DPYSL3*) (**Supplementary Figure 2A**). In both E3 and E4 cells, *PROX1* expression was up-regulated from D3 to D18, and then either did not change (E3) or was slightly decreased (E4) at D43. While it is difficult to make direct comparisons between snRNA-seq and bulk RNA-seq datasets, the lack of further up-regulation in *PROX1* at D43 in our bulk-level dataset could be related to the finding that *PROX1* is expressed across various stages of DGC maturation (hence, being a pan-granule cell marker irrespective of maturation stage) (Zhou et al., 2022). Interestingly, *CALB2* in E3 cells of our bulk RNA-seq dataset also showed an increased expression from D3 to D18 with no further change between D18 and D43; while E4 had an almost linear increased expression from D3 to D43. *NEUROD6* was clearly up-regulated at D43 compared to previous timepoints, while *DPYSL3* expression was stable from D3 to D43, regardless of *APOE* genotype. As mentioned above, comparisons between snRNA-seq and bulk RNA-seq data should be made with caution. Nevertheless, our interim conclusion is that our *in vitro* model is likely to have generated a heterogeneous population of DGCs, and a more in-depth analysis method (such as single-cell or snRNA-seq) will enable us to directly examine each of these populations at greater depth.

We next examined genes differentially expressed in human immature DGCs versus mature DGCs: according to Zhou and colleagues’ dataset, Calbindin 1 (*CALB1*) was more enriched in mature DGCs, whereas immature DGCs were characterised with higher expression of *DCX* and *STMN1*. D43 cells in our *in vitro* model had lower expression of *CALB1* than *DCX* and *STMN1*, irrespective of *APOE* genotype (**Supplementary Figure 2B**). We also analysed other genes from Zhou and colleagues' dataset that distinguished human immature DGCs from mature DGCs. For example, Protocadherin 20 (*PCDH20*) and *ROBO2* were more highly expressed in immature DGCs, while Synaptoporin (*SYNPR*) and EPH Receptor A6 (*EPHA6*) were enriched in mature DGCs in Zhou and colleagues’ dataset. D43 cells in our model had higher expression of *ROBO2* and *SYNPR* compared to *PCDH20* and *EPHA6* (**Supplementary Figure 2C**), which suggests a mixture of immature and mature DGC population in our *in vitro* model.

Taken together, the model used in this study is likely to have been composed of cells at variable stages of maturation, but the stark contrast between *CALB1* vs *DCX* and *STMN1* suggest that D43 cells were likely to have been ‘predominantly immature’ DGCs. As mentioned above, a more in-depth analysis method such as single-cell or snRNA-seq would allow us to address the question at greater depth.

Interestingly, we found a number of Stathmin family genes, such as Stathmin 2 (*STMN2*), Stathmin 3 (*STMN3*), and Stathmin 4 (*STMN4*), to be highly up-regulated at D43 in our dataset. The ‘slope’ of increase between D3 and D18 was steeper in *STMN2* and *STMN4*, compared to *STMN1* and *STMN3* (**Supplementary Figure 2D**). The up-regulation of other Stathmin family genes is partially in line with Zhou and colleagues’ study, which also shows *STMN3* and *STMN4* in the list of genes enriched in human immature DGCs (Supplementary Table 6 in their study). Furthermore, *STMN2* was also identified by Franjic and colleagues to be highly expressed in human DG neuroblasts (Figure 2C in their study). *STMN1* and *STMN3* were previously shown to be expressed across various human tissues, while *STMN2* and *STMN4* seem to be more specific to the nervous system (Bièche et al., 2003). It would be interesting to ascertain whether *STMN3* and *STMN4* are also more enriched in immature DGCs compared to mature DGCs, like *STMN1* (Zhou et al., 2022) and *STMN2* (Franjic et al., 2022).

## Supplementary References

Bièche, I., Maucuer, A., Laurendeau, I., Lachkar, S., Spano, A. J., Frankfurter, A., . . . Curmi, P. A. (2003). Expression of stathmin family genes in human tissues: non-neural-restricted expression for SCLIP. *Genomics*, *81*(4), 400-410. <https://doi.org/10.1016/s0888-7543(03)00031-4>

Franjic, D., Skarica, M., Ma, S., Arellano, J. I., Tebbenkamp, A. T. N., Choi, J., . . . Sestan, N. (2022). Transcriptomic taxonomy and neurogenic trajectories of adult human, macaque, and pig hippocampal and entorhinal cells. *Neuron*, *110*(3), 452-469.e414. <https://doi.org/10.1016/j.neuron.2021.10.036>

Zhou, Y., Su, Y., Li, S., Kennedy, B. C., Zhang, D. Y., Bond, A. M., . . . Song, H. (2022). Molecular landscapes of human hippocampal immature neurons across lifespan. *Nature*, *607*(7919), 527-533. <https://doi.org/10.1038/s41586-022-04912-w>
